# Direct Comparison of the Prediction of the Unbound Brain-to-Plasma Partitioning Utilizing Machine Learning Approach and Mechanistic Neuropharmacokinetic Model

**DOI:** 10.1208/s12248-021-00604-x

**Published:** 2021-05-18

**Authors:** Yohei Kosugi, Kunihiko Mizuno, Cipriano Santos, Sho Sato, Natalie Hosea, Michael Zientek

**Affiliations:** 1 Global DMPK, Takeda California Inc., San Diego, California 92121 USA; 2 Global DMPK, Takeda Pharmaceutical Company Limited, 26-1 Muraoka-Higashi, 2-Chome, Fujisawa, Kanagawa 251-8555 Japan

**Keywords:** BCRP, brain-to-plasma unbound concentration ratio, *in silico*, machine learning, MDR1

## Abstract

The mechanistic neuropharmacokinetic (neuroPK) model was established to predict unbound brain-to-plasma partitioning (*K*_p,uu,brain_) by considering *in vitro* efflux activities of multiple drug resistance 1 (MDR1) and breast cancer resistance protein (BCRP). Herein, we directly compare this model to a computational machine learning approach utilizing physicochemical descriptors and efflux ratios of MDR1 and BCRP-expressing cells for predicting *K*_p,uu,brain_ in rats. Two different types of machine learning techniques, Gaussian processes (GP) and random forest regression (RF), were assessed by the time and cluster-split validation methods using 640 internal compounds. The predictivity of machine learning models based on only molecular descriptors in the time-split dataset performed worse than the cluster-split dataset, whereas the models incorporating MDR1 and BCRP efflux ratios showed similar predictivity between time and cluster-split datasets. The GP incorporating MDR1 and BCRP in the time-split dataset achieved the highest correlation (*R*^2^ = 0.602). These results suggested that incorporation of MDR1 and BCRP in machine learning is beneficial for robust and accurate prediction. *K*_p,uu,brain_ prediction utilizing the neuroPK model was significantly worse compared to machine learning approaches for the same dataset. We also investigated the predictivity of *K*_p,uu,brain_ using an external independent test set of 34 marketed drugs. Compared to machine learning models, the neuroPK model showed better predictive performance with *R*^2^ of 0.577. This work demonstrates that the machine learning model for *K*_p,uu,brain_ achieves maximum predictive performance within the chemical applicability domain, whereas the neuroPK model is applicable more widely beyond the chemical space covered in the training dataset.

**Supplementary Information:**

The online version contains supplementary material available at 10.1208/s12248-021-00604-x.

## INTRODUCTION

One of the biggest impediments in drug discovery to accomplish favorable pharmacological effects for central nervous system (CNS) diseases is to design drugs with high blood-brain barrier (BBB) permeability (1, 2). Two active efflux transporters, multiple drug resistance 1 (MDR1) and breast cancer resistance protein (BCRP), expressed at the BBB, are mainly responsible for decreased brain concentration of drugs (3–7). Since higher unbound brain-to-plasma partitioning (*K*_p,uu,brain_) values are usually favorable to yield a lower dose and systemic exposure (1, 8), assessments of *K*_p,uu,brain_ are a key process in CNS drug discovery (9). The *K*_p,uu,brain_ is derived from not only *in vitro* binding in the plasma and brain homogenate, but also the *in vivo* brain to plasma concentration ratio derived in animal studies. These *in vivo* studies require significant resource, and also raise ethical issues. Therefore, a quantitative *K*_p,uu,brain_ prediction based on *in silico* and *in vitro* data consisting of *in vitro* cell line assessments is beneficial in reducing chemistry cycle times for chemical optimization.

Several machine learning approaches based on *in silico* data have been used for *K*_p,uu,brain_ prediction (10–15), and the predictivity was improved by incorporating the efflux activity of MDR1 as an explanatory variable (16). However, in a particular example by Dolgikh and colleagues using MDR1 expressing cells, which did not include BCRP efflux activity in the prediction, an influential aspect of their work is the source of the MDR1 cell line, where the authors used a cell line supplied by the Netherlands Cancer Institute. The cell line appears to be less sensitive compared to those established at National Institutes of Health (NIH), which would lessen the ability to derive accurate predictions (9, 17, 18). Hence, it is thought that there is room for further improvement in the predictivity of machine learning approaches by incorporating sensitive efflux activities of MDR1 cells from NIH and the additional activity of BCRP cell lines.

In addition to machine learning models, mechanistic neuroPK models considering efflux activities of MDR1 and/or BCRP are also powerful tools to predict *K*_p,uu,brain_ (17, 19–21). Mechanistic neuroPK models enable the translation across species through the use of relative activity factors (RAF) and relative expression factors (REF). These scaling factors bridge *in vitro* cell lines over-expressing transporters to tissue/cellular fractions by incorporating proteomic quantitation of the transporters and associating to activity (22). Both of these models have been useful for predicting human *K*_p,uu,brain_; however, the predictive ability of the neuroPK model has not been directly compared to machine learning approaches using the same dataset; therefore, it is still unclear which approach is more predictive, and better suited in particular situations.

This study directly compares the predictability of *K*_p,uu,brain_ predictions using both the neuroPK model and machine learning approach, which incorporates both the efflux transporter activities of MDR1 and BCRP across the same dataset.

## MATERIALS AND METHODS

### Materials

A total of 640 internal compounds were obtained from Takeda Pharmaceutical Company (Fujisawa, Japan). All other reagents and solvents were of analytical grade or better and were commercially available.

### Animals

All the experimental protocols and procedures were approved by the Institutional Animal Care and Use Committee of the Shonan Health Innovation Park, Takeda Pharmaceutical Company Ltd., and all the animal experiments were performed at an animal research facility in Shonan Health Innovation Park accredited by the Association for Assessment and Accreditation of Laboratory Animal Care International. Male Sprague-Dawley rats (7–9 weeks old) were purchased from Charles River Laboratories (Wilmington, MA).

### *In Vivo* Compound Administration and Sampling of Plasma and Brain

Plasma and brain concentrations of proprietary compounds after intravenous, subcutaneous, intraperitoneal, and oral administration were determined. At two time points after the administration (Supporting Information, Table S5), whole blood and whole brain were collected. The blood samples were collected in a heparinized tube from the abdominal aorta and immediately centrifuged to obtain plasma. Brain samples were homogenized immediately after collection in 4 times the volume of the brain using saline under ice-cold conditions. All samples were stored at − 80°C until analysis by liquid chromatography-tandem mass spectrometry (LC-MS/MS, Applied Biosystems, Foster City, CA, USA). Before conducting this analysis, samples were thawed on ice and mixed with three volumes of acetonitrile. The supernatants were diluted and injected into the LC-MS/MS system to quantify compound concentrations using a calibration standard curve.

### *In Vitro* Permeability MDR1 and BCRP-Expressing Cells

Test compound solubilized in dimethyl sulfoxide (DMSO) were added to transport buffer (Hanks’ balanced salt solution with 10 mM HEPES, pH 7.4) at a final concentration of 2 μM (DMSO < 1%), on either the apical or basolateral side of the transwell chamber with the Madin-Darby canine kidney (MDCK)-MDR1 from NIH and MDCK-BCRP from Solvo Biotechnology (Szeged, Hungary). The confluent cell monolayers on the transwell were incubated for 1 h at 37°C with 5% CO_2_. Test compounds were quantified by LC/MS/MS analysis. Permeation of the test compounds from apical to basolateral (A to B) direction or B to A direction and the efflux ratio were determined. The apparent permeability coefficient Papp (cm/s) was calculated by using the following equation:

1 $${P}_{\mathrm{app}}=\frac{\mathrm{d}{\mathrm{C}}_{\mathrm{r}}}{\mathrm{d}\mathrm{t}}\times \frac{V_{\mathrm{r}}}{A\times {C}_0}$$

where dC_r_/dt is the cumulative concentration of the compound in the receiver chamber as a function of time (μM/s); *V*_r_ is the volume of the solution in the receiver chamber (0.075 mL on the apical side, 0.25 mL on the basolateral side); *A* is the surface area for transport, i.e., 0.0804 cm^2^ for the area of the monolayer; and *C*_0_ is the initial concentration in the donor chamber (μM).

The efflux ratio (ER) was calculated using the following equation:

2 $$\mathrm{ER}=\frac{P_{\mathrm{app},\mathrm{B}\ \mathrm{to}\ \mathrm{A}}}{P_{\mathrm{app},\mathrm{A}\ \mathrm{to}\ \mathrm{B}}}$$

### Unbound Fractions in Brain and Plasma of Rats

The unbound fraction in rat plasma and brains for each compound was evaluated using the equilibrium dialysis method, details of which have been reported previously (17). Briefly, test compound solutions in DMSO were diluted in rat plasma or 20% (*w*/*v*) rat brain homogenate in 100 mM sodium phosphate buffer (pH 7.4) to a concentration of 1 μM. Dialysis was conducted against an equal volume of 10 mM phosphate buffer (plasma) and 100 mM sodium phosphate buffer (brain homogenate) at 37°C for 16–20 h with 8% CO_2_ (plasma) and without CO_2_ (brain homogenate). Both the plasma and the brain homogenate obtained from the apparatus were added to equal volumes of control buffer, and then mixed with three volumes of acetonitrile. After centrifugation, the supernatants were analyzed by LC-MS/MS.

The unbound fraction in the incubation mixture (*f*_u,plasma_) was calculated using the following equation:

3 $${f}_{\mathrm{u},\mathrm{plasma}}=\frac{\mathrm{compound}\ \mathrm{concentration}\ \mathrm{in}\ \mathrm{buffer}}{\mathrm{comopound}\ \mathrm{concentration}\ \mathrm{in}\ \mathrm{plasma}}$$

The unbound fractions in the brain (*f*_u,brain_) were calculated using the following equation:

4 $${f}_{\mathrm{u},\mathrm{brain}}=\frac{1}{D\times \left(\frac{1}{{f_{\mathrm{u},\mathrm{brain}}}^{\prime }}-1\right)+1}$$

where *D* and *f*_u,brain_′ represent the dilution factor for the brain homogenate and unbound fraction determined in the 20% (*w*/*v*) brain homogenate, respectively.

### Determination of *K*_p,uu,brain_

After determination of concentration in plasma (*C*_plasma_) and brain (*C*_brain_), *K*_p,uu,brain_ was calculated by using the following equation:

5 $${K}_{\mathrm{p},\mathrm{uu},\mathrm{brain}}=\frac{f_{\mathrm{u},\mathrm{brain}}\times {C}_{\mathrm{brain}}}{f_{\mathrm{u},\mathrm{plasma}}\times {C}_{\mathrm{p}\mathrm{lasma}}}$$

### Prediction of *K*_p_,_uu,brain_ in Rats Based on NeuroPK Model

The neuroPK model used in this study was developed in a previous study (17). The major assumptions of this model are that the drug penetration is at steady state, active transport is governed only by two efflux transporters (whose flux is measured *in vitro* as ER_MDR1_ and ER_BCRP_), drug flux from bulk flow is minimal *in vivo*, and paracellular diffusion is absent *in vitro*. Based on these assumptions, *K*_p,uu,brain_ can be described using the following equation:

6 $${K}_{\mathrm{p},\mathrm{uu},\mathrm{brain}}=\frac{1}{1+\alpha \times \left({\mathrm{ER}}_{\mathrm{MDR}1}-1\right)+\beta \times \left({\mathrm{ER}}_{\mathrm{BCRP}}-1\right)}$$

where *α* represents a scaling factor of the *in vitro* efflux activity of MDR1 in MDCK cells against that of MDR1 *in vivo* and *β* represents a scaling factor of the *in vitro* efflux activity of BCRP in MDCK cells against that of BCRP *in vivo*. The detailed deviation method leading to Eq. 6 is discussed in two reported literature references (17, 20).

The parameters *α* and *β* in Eq. 6 were fitted simultaneously to the observed *K*_p,uu,brain_ values in rats using a nonlinear least-square methods provided by Curve Fitting Toolbox 3.5.7 equipped with Matlab R2018a (Mathworks Inc.).

### Model Building Using Machine Learning

Each prediction model was generated with StarDrop (StarDrop v6.5.0, Optibrium Ltd, Cambridge, UK) according to the previously described method (23, 24). StarDrop uses 2D SMARTS-based descriptors, which are counts of atom types and functionalities, along with whole molecule properties such as molecular weight (M.W.), topological polar surface area (TPSA), and logP (for a total of 330 descriptors). The descriptors were scaled to unit variance and mean-centered on zero. The rules for descriptor exclusion were as follows:

- Descriptors with a standard deviation less than 0.0005,
- Descriptors represented by less than 4% of compounds, and
- If the pair-wise correlation between any two descriptors exceeds 0.95, then the descriptor of the pair with the lowest correlation with the *Y* column is excluded.

All *in silico* models were built using random forest regression (RF) and Gaussian process (GP) model. RF is a flexible, easy to use machine learning algorithm that produces a great result frequently, even without hyper-parameter tuning (25, 26). We used 400 random forest trees for each model. The concept and detailed implementation of the GP method for regression problems were described in Obrezanova *et al*. (27). Briefly, 4 hyperparameters, θ1, θ2, θ3, and γi (i = 1…K), were optimized. The overall scale for the property values is given by θ1, and the γi are a set of length scale parameters, one for each descriptor. An overall constant shift in the function away from zero is given by θ2. The variance of the assumed noise in the data is described by hyperparameter θ3. A small value on the γi means that differences in the corresponding descriptor influence property values greatly. Hyperparameter tuning of γi was conducted by conjugate gradient optimization (GPOPT) (28). The descriptors ranked in the top 20 of feature importance used in GPOPT model for *K*_p,uu,brain_ prediction are summarized in Table S4 and Figure S4.

### Model Evaluation

Predictive performance in each model was assessed based on the coefficient of determination (*R*^2^) values and residual mean squared error (RMSE) as the statistical indexes. *R*^2^ and RMSE were calculated by the following equations:

7 $${R}^2=1-\frac{\sum_{k=1}^n{\left(\ Observed\ value- Predicted\ value\right)}^2}{\sum_{k=1}^n{\left( Observed\ value- Mean\ value\right)}^2}$$

8 $$\mathrm{RMSE}=\sqrt{\frac{1}{n}\times {\sum}_{k=1}^n{\left( Predicted\ value- Observed\ value\right)}^2}$$

where *n* represents the size of the dataset and *k* represents kth data; the resulting RMSE depicts the magnitude of difference from the observed value. *R*^2^ and RMSE were calculated using log(*K*_p,uu,brain_) and log(ER). The percentage of correct answers was evaluated using the number of predicted values against observed values within 2-fold variabilities. Paired *t* tests of squared errors were used to assess statistical differences between machine learning and the neuroPK models.

Three validation approaches were applied to investigate the model performance. In the first method, the data set is clustered using fingerprints of the molecule structures with a specified Tanimoto coefficient (29), and then split into training and test set with a ratio of 80:20 depending on the property value. This approach enables us to estimate the predictivity for the case that compounds with similar scaffold were included in training and test data set. In the second method, time-split validation was applied to estimate the predictivity for new chemical entities that chemists are likely to investigate in the future (30). The total data set was split by date of assay, i.e., 80% of the total data set before a certain date were assigned as the time-split training set while the remaining 20% of data after that date were assigned as the time-split test set. In the third method, 34 commercially available compounds were used to assess the model performance as an external dataset.

## RESULTS

### Dataset Analysis

**Fig. 1:**
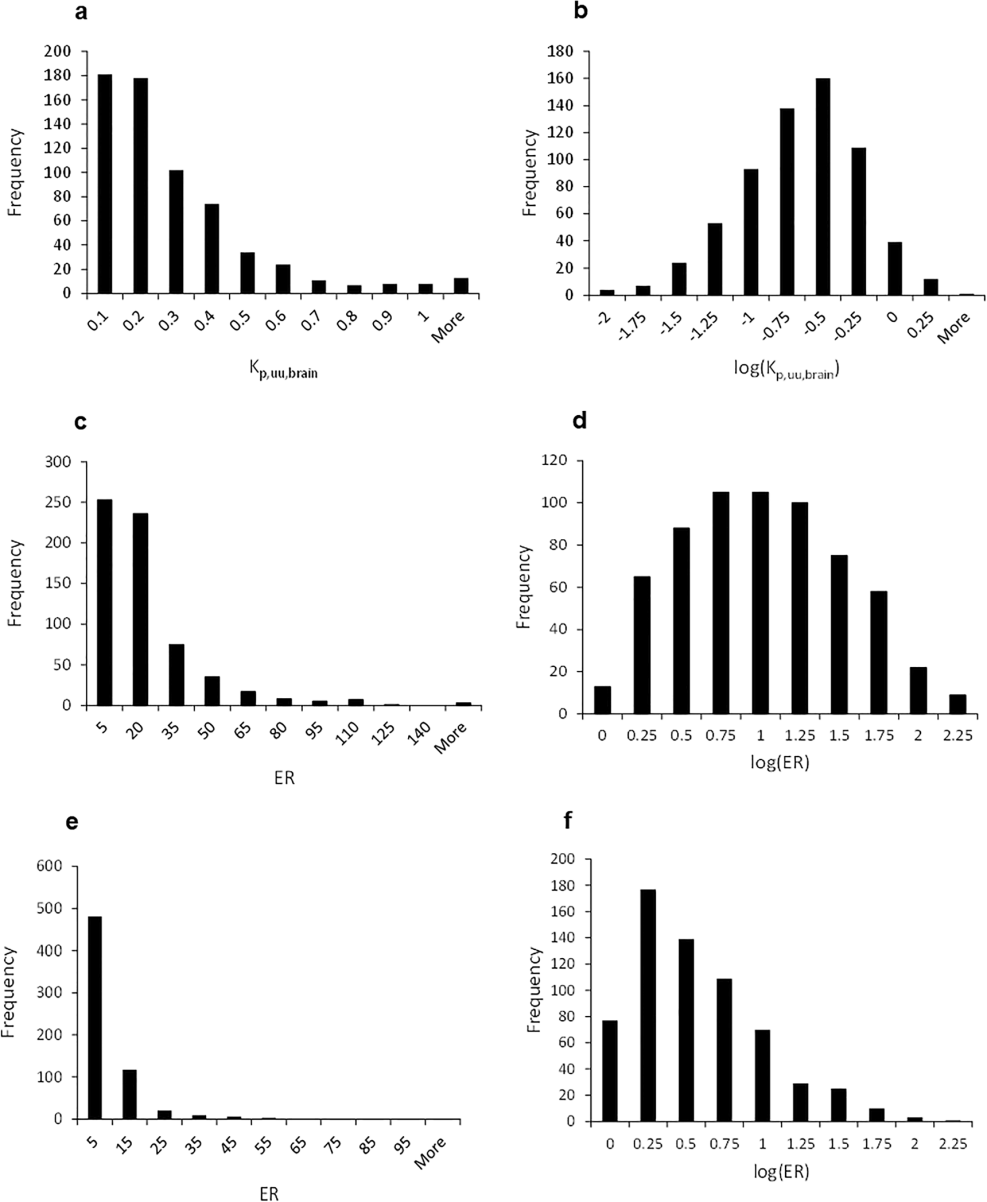
Distribution of **a** *K*_p,uu,brain_, **b** log(*K*_p,uu,brain_), **c** ER, and **d** log(ER) in MDR1 cells, **e** ER and **f** log(ER) in BCRP cells for 640 compounds

*In vivo K*_p_, *in vitro* brain tissue and plasma protein binding, and efflux activities of MDR1 and BCRP were experimentally determined for 640 proprietary compounds. This study covered a broad chemical diversity representing a wide range of physicochemical properties, ER and *K*_p,uu,brain_ (Supporting Information, Figure S1 and S2, M.W., 187 to 555; clogP, 0.291 to 5.20; TPSA, 12.0 to 157; ER in MDR1 cells, 0.563 to 151; ER in BCRP cells, 0.522 to 100; *K*_p,uu,brain_, 0.00354 to 2.82). Data of both *K*_p,uu,brain_ and ER in MDCK-MDR1 from NIH cells and MDCK-BCRP cells did not follow the normal distribution and were log transformed to reduce unequal error variances (Fig. 1). The cluster-split training and test sets showed similar distributions of log(*K*_p,uu,brain_) (Supporting Information, Figure S1), indicating that the splitting method adopted was reasonable. However, M.W., clogP, and TPSA in the time-split test sets tended to have higher values than those in the time-split training sets (Supporting Information, Figure S2). The *K*_p,uu,brain_ at the earlier time point was within 2-fold of that at the later point for 95.0% of the compounds (Supporting Information, Figure S3), and therefore, *K*_p,uu,brain_ was assumed to be determined under steady-state conditions. *K*_p,uu,brain_ values at the later time point were used for model building.

### Prediction of Efflux Activities of MDR1 and BCRP Using Machine Learning Approach

**Fig. 2:**
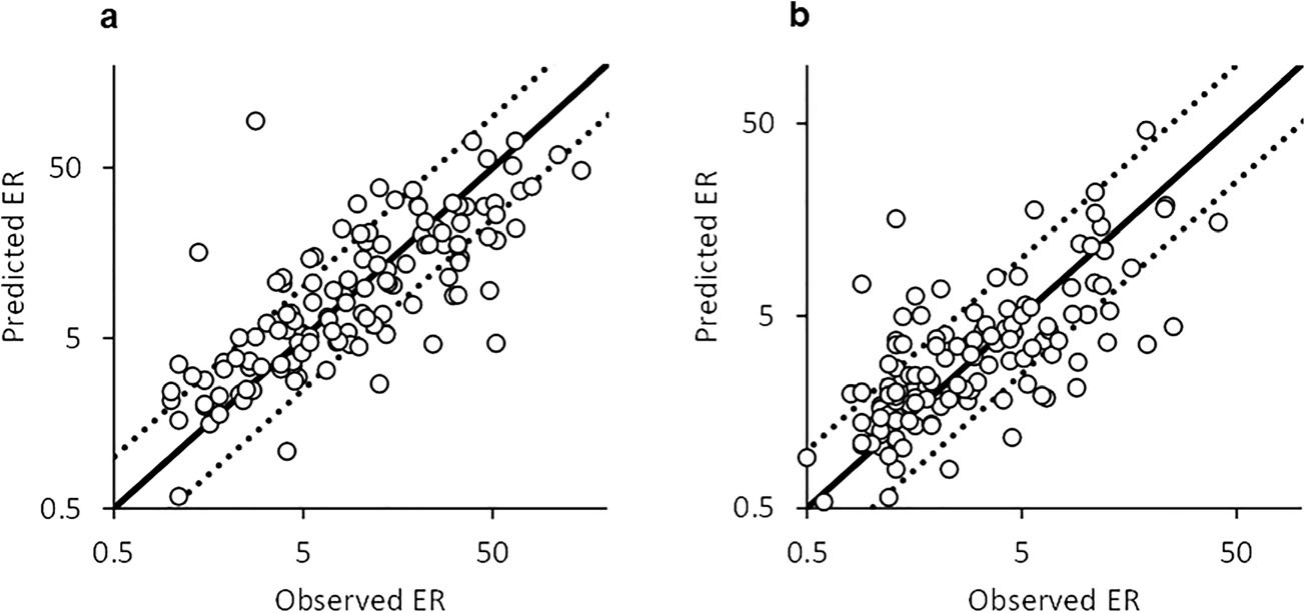
Comparison of the observed **a** MDR1 and **b** BCRP efflux ratio with values predicted by GPOPT in the cluster-split test set. Each figure represents results of 128 compounds in the test set. Solid line is the line of unity. Dashed lines indicate 2-fold deviation

Transcellular transport activities characterized using MDCK-MDR1 cells from NIH and MDCK-BCRP cells were used to develop the machine learning model. Among tested compounds, 84.4% of compounds had efflux ratios > 2 in MDR1 assay and 56.1% for BCRP. The machine learning models for MDR1 and BCRP developed by GPOPT showed *R*^2^ of 0.581 and 0.499, respectively, in the cluster-split test set (Table I). The prediction of efflux activities in the cluster-split dataset also indicated that 71.1% and 75.0% of those compounds in MDR1 and BCRP models, respectively, were predicted within 2-fold of the observed values (Fig. 2). The predictivity of the machine learning models in the time-split dataset was less than that of the cluster-split dataset, suggesting that the prediction models for efflux activities may not be applied to chemotypes different from compounds included in the training set. In particular, the model for BCRP showed poor prediction. Therefore, *K*_p,uu,brain_ prediction using predicted efflux ratio for MDR1 and BCRP was conducted only in the cluster-split test set (Supporting Information, Table S1).

**Table I:** Predictive Performance of Efflux Activities of MDR1 and BCRP in Cluster and Time-Split Test Set by GPOPT

		MDR1	BCRP
Cluster split	% < 2-fold	71.1	75.0
	*R*^2^	0.581	0.499
	RMSE	0.333	0.294
Time split	% < 2-fold	50.8	46.9
	*R*^2^	0.317	0.070
	RMSE	0.490	0.558

*R*^2^ and RMSE were calculated using log(ER)

### Prediction of *K*_p,uu,brain_ Using Machine Learning Approaches

**Fig. 3:**
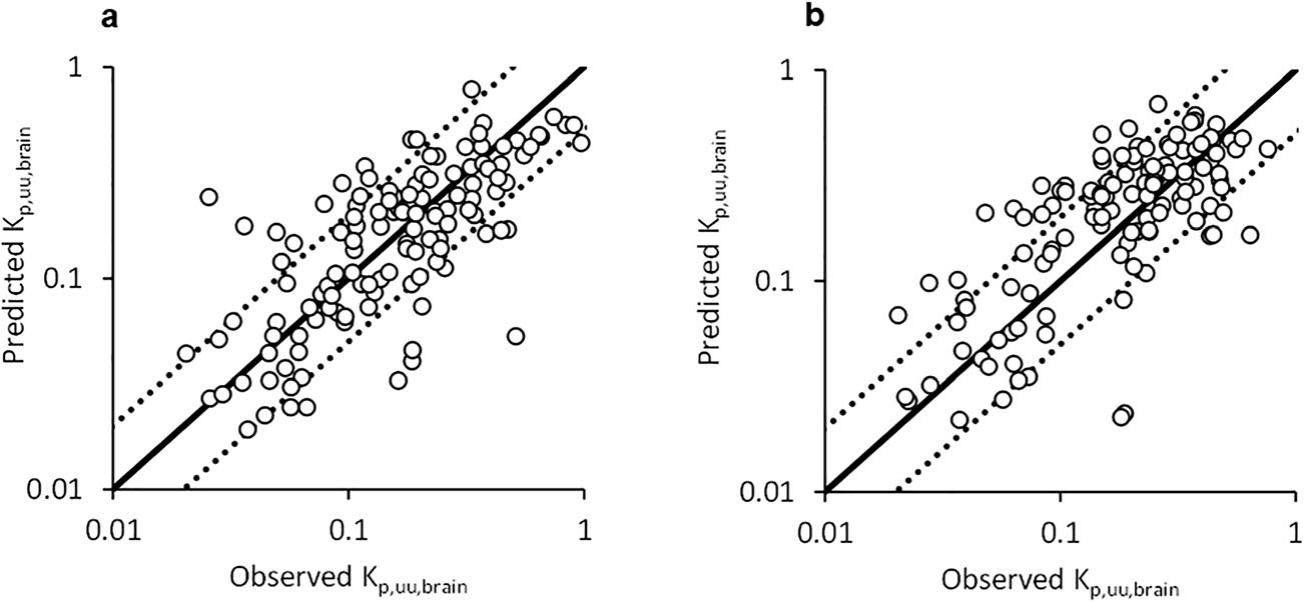
Observed *K*_p,uu,brain_ versus values predicted by GPOPT incorporating MDR1 and BCRP efflux ratio in **a** cluster-split and **b** time-split test set. Each figure represents results of 128 compounds in the test set. Solid line is the line of unity. Dashed lines indicate 2-fold deviation

The log(*K*_p,uu,brain_) values were predicted by RF and GPOPT. For the determination of the percentage of compounds falling within the 2-fold variability, *K*_p,uu,brain_ was transformed from predicted log(*K*_p,uu,brain_) to compare with the neuroPK model that directly predicted *K*_p,uu,brain_ based on Eq. 6 (Fig. 3 and Table II). The predictivity based on *R*^2^ and RMSE was improved by the incorporation of MDR1 and BCRP efflux activities in both cluster and test-split datasets. Overall, MDR1 showed the greatest impact on the predictive performance of *K*_p,uu,brain_ compared to the BCRP addition. The highest *R*^2^ of 0.602 was obtained by using GPOPT and both MDR1 and BCRP activities were taken into account pertaining to the time-split dataset with 73.4% of the compounds being predicted within 2-fold of the observed value (Fig. 3b). When leaving the MDR1 and BCRP activities out of the prediction, the cluster-split approach resulted in superior performance compared to the time-split approach (Table II). Meanwhile, the time-split approach incorporating both the MDR1 and the BCRP activities performed equally to the cluster-split approach. The top 20 features obtaining the lowest length scale were extracted in RF and GPOPT models incorporating both MDR1 and BCRP activities (Supporting Information, Figure S4 and Table S4). Efflux ratios of MDR1 and BCRP were ranked in the top 20 of feature importance.

**Table II:** Predictive Performance of *K*_p,uu,brain_ in Cluster and Time-Split Test Set by RF and GPOPT

Efflux ratio		MDR1	Not used	*In vitro*	Not used	*In vitro*
		BCRP	Not used	Not used	*In vitro*	*In vitro*
RF	Cluster split	% < 2-fold	68.0	74.2	69.5	76.6
		*R*^2^	0.363	0.427	0.389	0.489
		RMSE	0.310	0.294	0.303	0.277
	Time split	% < 2-fold	64.8	67.2	66.4	74.2
		*R*^2^	0.227	0.335	0.318	0.504
		RMSE	0.371	0.348	0.354	0.311
GPOPT	Cluster split	% < 2-fold	69.5	78.1	73.4	77.3
		*R*^2^	0.422	0.563	0.454	0.536
		RMSE	0.297	0.267	0.297	0.286
	Time split	% < 2-fold	49.2	75.8	60.2	73.4
		*R*^2^	0.307	0.518	0.427	0.602
		RMSE	0.422	0.301	0.377	0.292

*R*^2^ and RMSE were calculated using log(*K*_p,uu,brain_)

### Prediction of *K*_p,uu,brain_ Using Translational NeuroPK Model

**Fig. 4:**
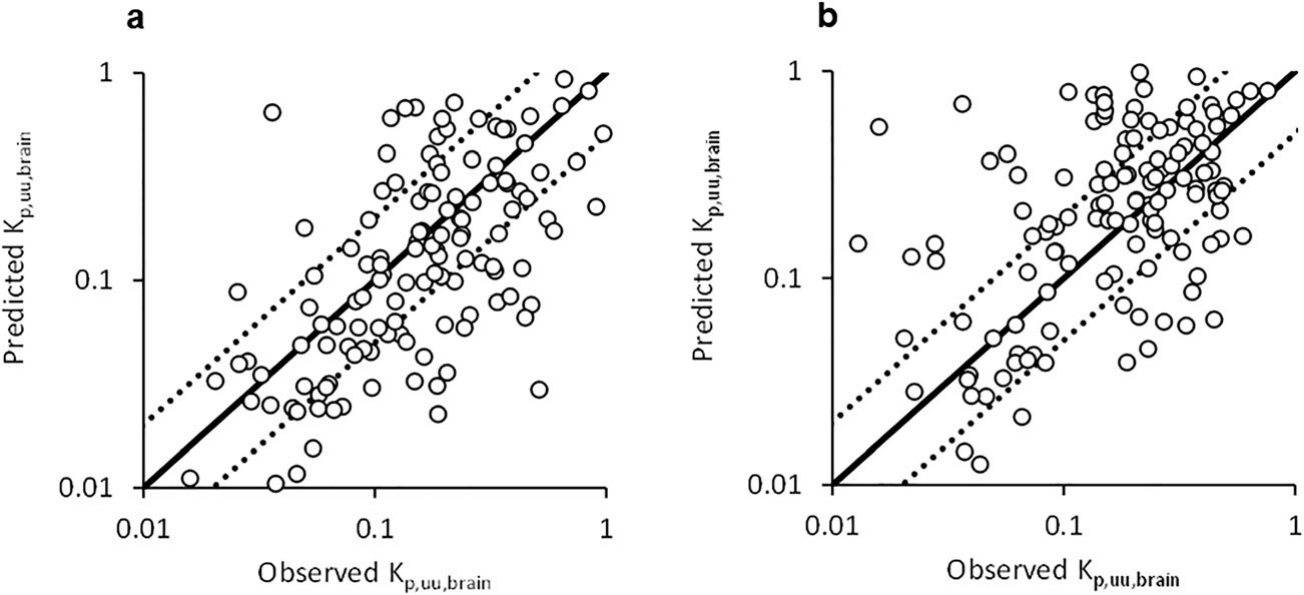
Comparison of the observed *K*_p,uu,brain_ and values predicted by the neuroPK model in **a** cluster-split and **b** time-split test set. Each figure represents results of 128 compounds in the test set. Fitting was performed to estimate scaling factors *α* and *β* against observed values of *K*_p,uu,brain_ in rats. Solid line is the line of unity. Dashed lines indicate 2-fold deviation

By using the neuroPK model, scaling factors for MDR1 (*α*) and BRCP (*β*) were estimated by fitting against *K*_p,uu,brain_ (Table III). Using the model incorporating both efflux transporter activities, 58.6% of compounds were predicted within 2-fold of the observed *K*_p,uu,brain_ value with a trending *R*^2^ value of 0.386 in cluster test sets (Table III and Fig. 4). Meanwhile, the time-splitting approach resulted in a lower *R*^2^ value of 0.265. *K*_p,uu,brain_ prediction utilizing the neuroPK model was significantly worse compared to machine learning approaches for the same dataset (*p* < 0.01). The models incorporating *in silico* MDR1 and BCRP showed an even lower predictive performance than that using experimental values with an *R*^2^ value of 0.268 in the cluster test set (Supporting Information, Table S2).

**Table III:** Predictive Performance of *K*_p,uu,brain_ in Cluster and Time-Split Test Set by the NeuroPK Model

	*α* [95% Cl]	0.58 [0.44, 0.71]
	*β* [95% Cl]	1.2 [0.75, 1.6]
Cluster split	% < 2-fold	58.6
	*R*^2^	0.386
	RMSE	0.396
	*α* [95% Cl]	0.36 [0.25, 0.46]
	*β* [95% Cl]	1.8 [1.3, 2.3]
Time split	% < 2-fold	57.8
	*R*^2^	0.265
	RMSE	0.434

*R*^2^ and RMSE were calculated using log(*K*_p,uu,brain_)

### Model Comparison Using External Dataset

The MDR1 and BCRP activities were determined for 34 commercially available compounds in which *K*_p,uu,brain_ values were reported by Friden *et al*. (11), Summerfield *et al*. (31), Kodaira *et al*. (32), and our previous research (17). In this external test set for the established models, 18 and 10 of 34 compounds were substrates of MDR1 and BCRP based on *in vitro* assessment, respectively, with efflux ratios > 2 (Supporting Information, Table S3). We then applied the machine learning models and the neuroPK model established by the 640 compound internal data to the external test set. The *R*^2^ in neuroPK model was 0.577, which was higher than those of 0.422 and 0.363 in GP and RF, respectively (Table IV).

**Table IV:** Predictive Performance of *K*_p,uu,brain_ in External Test Set

	Neuro PK	RF	GPOPT
% < 2-fold	44.1	38.2	44.1
*R*^2^	0.577	0.434	0.479
RMSE	0.542	0.609	0.556

*R*^*2*^ and RMSE were calculated using log(*K*_p,uu,brain_)

## DISCUSSION

It has been reported that MDCK-MDR1 cells obtained from NIH provide a more sensitive *in vitro* tool for determining MDR1-mediated efflux liability than MDCK-MDR1 from the Netherlands Cancer Institute, or MDR1-overexpressing Lilly Laboratories cell porcine kidney 1 cells (LLC-PK1-MDR1) (9, 18). Previously, we also reported that the NIH cell line MDCK-MDR1 was superior as an MDR1-overexpressing cell line to LLC-PK1-MDR1 for quantitatively predicting brain disposition (17). This work is the first report to establish machine learning models using efflux activity data of MDCK-MDR1 from the NIH-supplied cell line. In addition, there are few reports about quantitative prediction of the efflux transporter BCRP by *in silico* modeling methods, although several qualitative classification models have been reported (33–39). The *in vitro* prediction results of the MDR1 and the BCRP efflux assays indicate that the predictivity of MDR1 efflux activity was higher than the predictivity of BCRP efflux activity (Table I). Based on the results, this difference in predictivity appears to be caused by the differences of distribution patterns, which log(ER) in MDR1 showed relatively higher than that in BCRP cells (Fig. 1d and f). The predictivity in the time-split dataset was worse than that in the cluster-split dataset, suggesting that *in silico* MDR1 and BCRP models are more applicable to compounds with similar structure, or with timely updating of the model for practical use.

The efflux activities predicted by *in silico* MDR1 and BCRP models were used for the *K*_p,uu,brain_ prediction as additional explanatory variables (Supporting Information, Table S1). *In silico* predictions of MDR1 and BCRP efflux resulted in a limited predictivity of the machine learning model for *K*_p,uu,brain_, suggesting that current dataset scale and predictivity for MDR1 and BCRP models are not sufficient to apply the *K*_p,uu,brain_ prediction. Meanwhile, *in silico* approaches, underpinned by *in vitro* assessments, can utilize larger datasets than that by *in vivo* studies in early discovery, and therefore, the predictivity will be improved when larger training sets are employed. Indeed, Dolgikn *et al*. succeeded in improving the predictive performance of their model for *K*_p,uu,brain_ by using an internal MDR1 model trained with a significantly larger dataset (16).

Although several *in silico K*_p,uu,brain_ prediction models have been reported (10–16), there are few *K*_p,uu,brain_ prediction models considering experimentally determined MDR1 and/or BCRP activities. This study revealed that the predictive performance of *K*_p,uu,brain_ tended to be improved by considering BCRP activities in both GPOPT and RF models (Table II), suggesting that the BCRP activity also contributes in part to the predictivity of *K*_p,uu,brain_. However, the predictive performance using only MDR1 activity was better than that of using the BCRP activity alone in both the cluster and time-splinting datasets. Considering that the expression level of Mdr1a has been shown to be higher than Bcrp in brain capillaries of the rat (40), it therefore seems reasonable that the substrates for MDR1 would have a larger impact on the *K*_p,uu,brain_ assessments compared to BCRP.

In this study, the machine learning models were based on rational selection using the clustering approach, which often provided an optimistic prediction result compared to the time-split approach. The authors believe this is due in part to the addition of new chemical scaffolds being added over time (30, 41–43). Indeed, the models developed by only *in silico* descriptors showed higher predictivity in the cluster-split dataset than that in the time-split dataset (Table II), suggesting that practical operation of a machine learning model warrants timely model updates to cover new chemical space. Interestingly, the machine learning models incorporating MDR1 and BCRP activities showed similar predictivity between the time-split and the cluster-split datasets. This result indicated that the drawback of the machine learning model could be resolved by considering MDR1 and BCRP activities as an explanatory variable.

In the neuroPK model, the *α* and *β* in this study were lower than those in previous studies using *K*_p,brain_ ratio obtained from *K*_p,brain_ in wild-type and Mdr1a (−/−)/Bcrp(−/−) (dual KO) rats even though the same MDR1 and BCRP cell lines were used. Although the *K*_p,brain_ ratio is reported to be a direct index of *K*_p,uu,brain_ based on the effect of the *in vivo* MDR1 and BCRP expressed at the BBB (21), actual *K*_p,brain_ ratios tend to be higher than *K*_p,uu,brain_ (17). Thus, the difference between *K*_p,brain_ ratio and *K*_p,uu,brain_ would lead to different *α* and *β* correction factors. When setting *α* and *β* in the neuroPK model, consideration of both efflux activities in cell lines and predictive endpoints are necessary for accurate prediction.

While the time-split approach using in-house data is most useful in estimating the predictive performance of ongoing internal drug discovery programs, the authors also investigated whether the established model was applicable to external compounds (Table IV). Out of 34 external compounds, 44.1% compounds were predicted to within 2-fold of the observed values in the GPOPT and the neuroPK models. Meanwhile, based on *R*^2^ and RMSE, the neuroPK models showed better prediction performance compared to machine learning models. The distribution of M.W., clogP, and TPSA in the external test set is less consistent with the external test set (Supporting Information, Figure S5), indicating that the coverage of a chemical space is different between internal and external datasets. These results suggested that the neuroPK model is widely applicable over structurally diverse datasets, while machine learning would achieve maximum performance for predicting compounds with similar chemical properties to those used in the compound training set.

Another advantage of the neuroPK model is the utilization of MDR1 and BCRP variables to establish *in vitro* to *in vivo* correlations (IVIVE) to be used in translation (22). These are presented by *α* and *β*, in the aforementioned equations. RAF approach corresponding to the ratio of *in vitro* to *in vivo* efflux activities for MDR1 and BCRP and REF approach using the ratio of transporter protein levels between *in vitro* cell lines and *in vivo* BBB are applicable to estimate these variables across the species. In comparison, the machine learning model suggests that large datasets are required to build accurate models. Therefore, building an inclusive model is limited by the lack of species-specific data, such as non-human primate and human. In order to estimate the minimum number of data required for the machine learning model, the model performance was investigated by changing the number of compounds used for training via the cluster-split dataset (Supporting Information, Figure S6). This optimistic assessment suggested that more than 50 data points were required to obtain equal to or greater predictivity compared to the neuroPK model. Another way to extend the *K*_p,uu,brain_ prediction model from rat to other species is that efflux activities in the rat model can be corrected by the ratio of *in vivo* transporter expression levels between rat and the other species. This hybrid approach could be applicable for both neuroPK and machine learnings models; however, additional validation is needed.

## CONCLUSIONS

This work has clarified the characterization of the neuroPK model and machine learning approaches for *K*_p,uu,brain_ prediction. Incorporating *in vitro* MDR1 and BCRP activities is useful to improve the predictivity and coverage of application by machine learning approaches for *K*_p,uu,brain_ prediction. Machine learning models have advantages for a homologous series of compounds in internal *K*_puu,brain_ prediction where sufficient data can be secured. Since the machine leaning approach requires a large dataset for model building, the neuroPK model is preferred in translation of *K*_p,uu,brain_ from rodent to monkey and human by considering RAF and REF approaches at this time. Additionally, the neuroPK model provides better predictivity of *K*_p,uu,brain_ for external compounds which are outside the chemical space in which the model was derived.

## Supplementary Information

ESM 1 (DOCX 657 kb)

## Abbreviations

MDR1: multiple drug resistance 1
BCRP: breast cancer resistance protein
GP: Gaussian processes
RF: random forest regression
CNS: central nervous system
BBB: blood-brain barrier
LLC-PK1: Lilly Laboratories cell porcine kidney 1
NIH: National Institutes of Health
M.W.: molecular weight
TPSA: topological polar surface area
