# Supplementary material for: Direct Comparison of the Prediction of the Unbound Brain-to-Plasma Partitioning Utilizing Machine Learning Approach and Mechanistic Neuropharmacokinetic Model
Source: AAPS J. 2021 May 18;23(4):72. doi: 10.1208/s12248-021-00604-x (PMC8131289; doi:10.1208/s12248-021-00604-x)
Supplement: Supplementary file 1 — (DOCX 657 kb) [file 12248_2021_604_MOESM1_ESM.docx]

Direct Comparison of the Prediction of the Unbound Brain-to-Plasma Partitioning Utilizing Machine Learning Approach and Mechanistic Neuropharmacokinetic Model

Yohei Kosugi,* Kunihiko Mizuno, Cipriano Santos, Sho Sato, Natalie Hosea, and Mike Zientek.

Global DMPK, Takeda California Inc., San Diego, California 92121, United States

**Supporting Information**


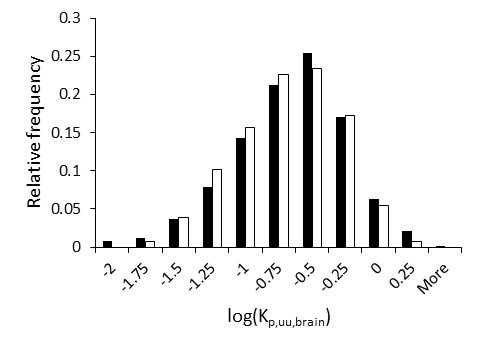

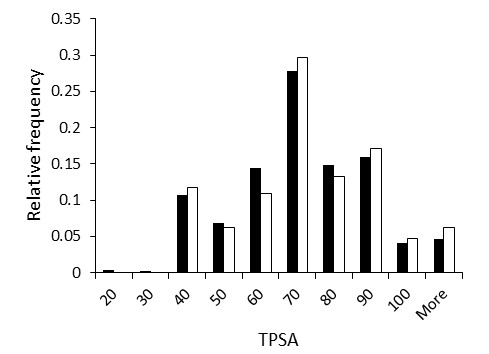


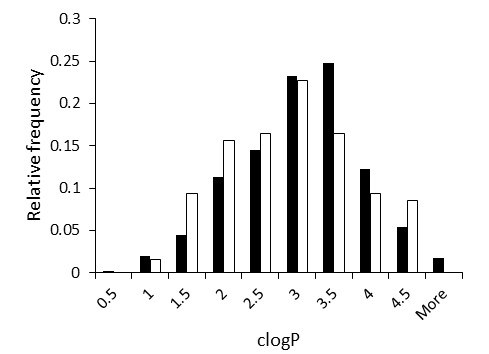

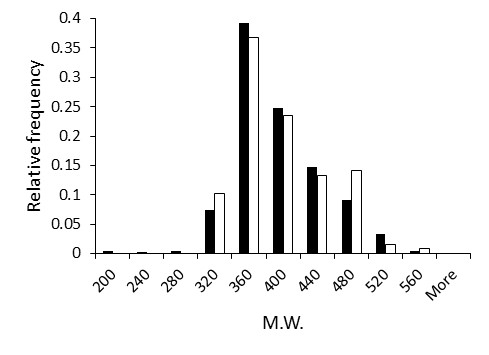


**
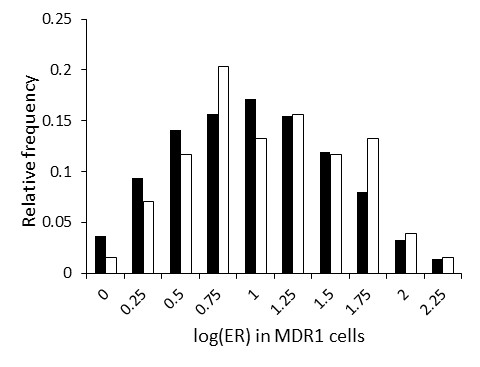

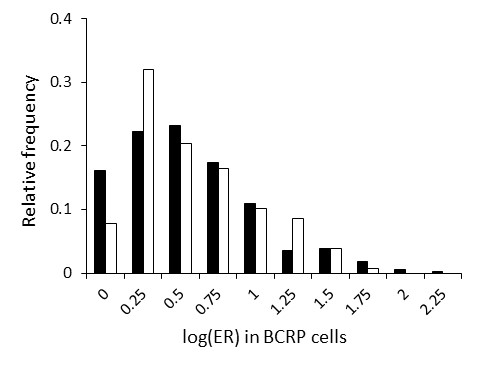
**

**Figure S1.** Distribution of log(K_p,uu,brain_) and physicochemical parameters, TPSA, clogP, M.W., log(ER) in MDR1 and BCRP cells between the cluster-split training set (solid column) and the test set (open column). TPSA, clogP and M.W. were calculated by StarDrop. The relative frequency was calculated by dividing a frequency count by sum of all frequencies. The number of compounds in training and test sets was 512 and 128, respectively.


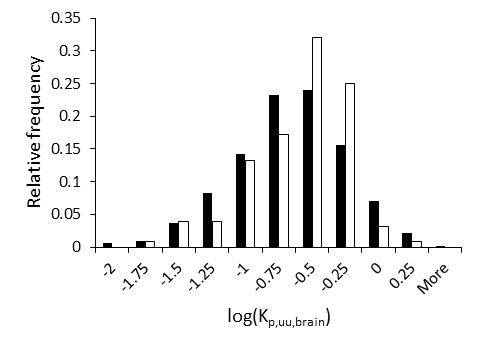

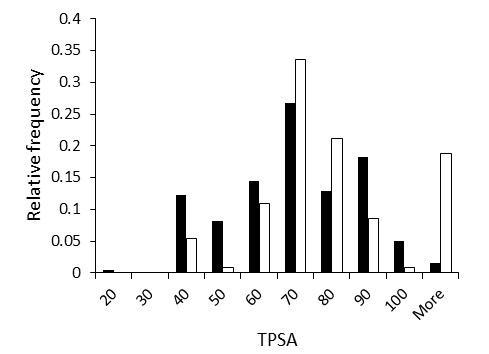


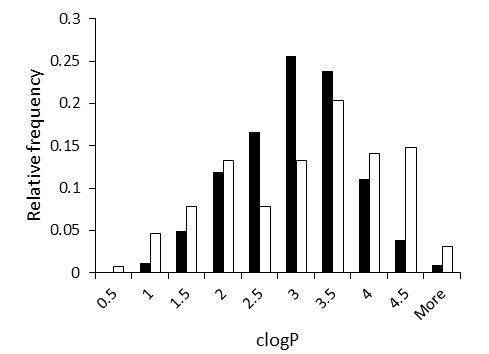

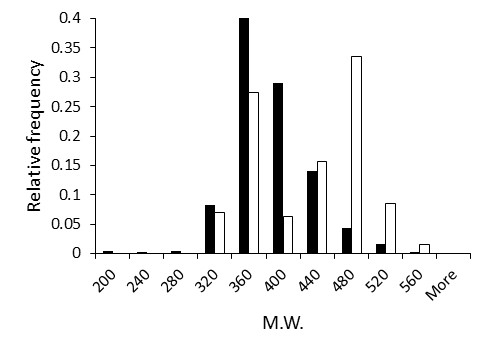


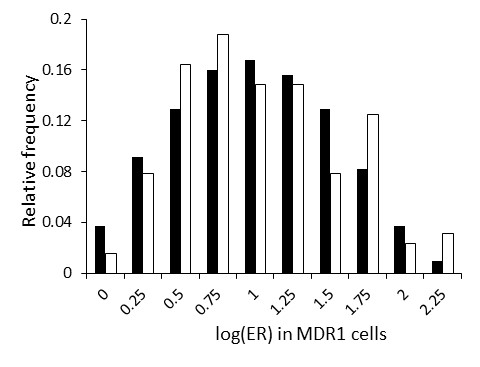

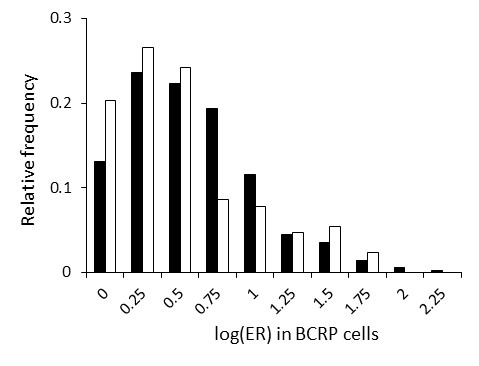


**Figure S2.** Distribution of log(K_p,uu,brain_) and physicochemical parameters, TPSA, clogP, and M.W. between the time-split training set (solid column) and the test set (open column). TPSA, clogP, M.W., log(ER) in MDR1 and BCRP cells were calculated by StarDrop. The relative frequency was calculated by dividing a frequency count by sum of all frequencies. The number of compounds in training and test sets was 512 and 128, respectively.


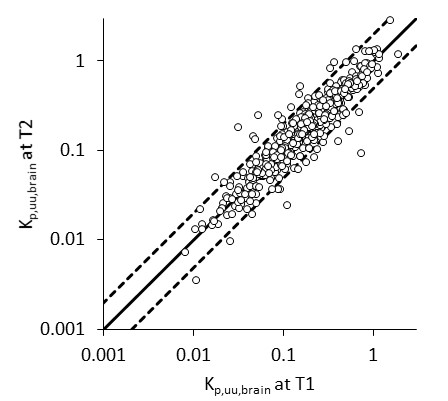


**Figure S3.** Comparison of the K_p,uu,brain_ for 640 compounds between two time points in rats. Solid black line indicates unity. Dashed lines on either side of the unity line represent factors of two-fold.

**a b**


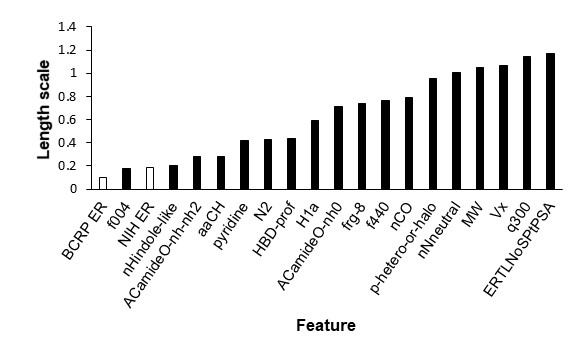
 **
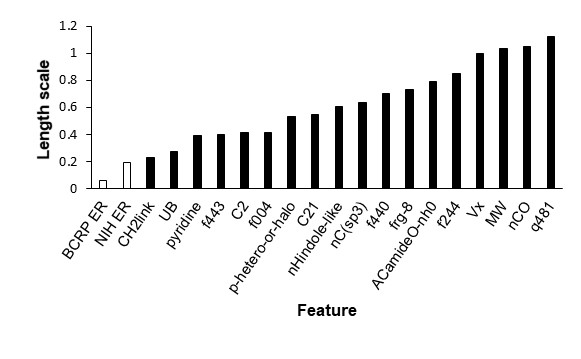
**

**Figure S4.** Feature importance analysis of the prediction of K_p,uu,brain_ for the top 20 features of GPOPT on (a) cluster-split, and (b) time-split training set. The normalized length scale was used as an indicator of feature importance. A small value on the normalized length scale means that differences in the corresponding descriptor influence property values very much. The open column indicates in vitro data. The solid column indicates 2D SMARTS-based descriptors.


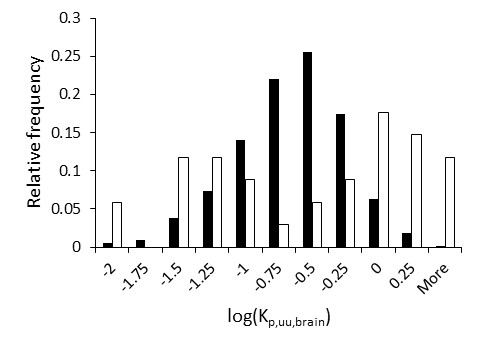

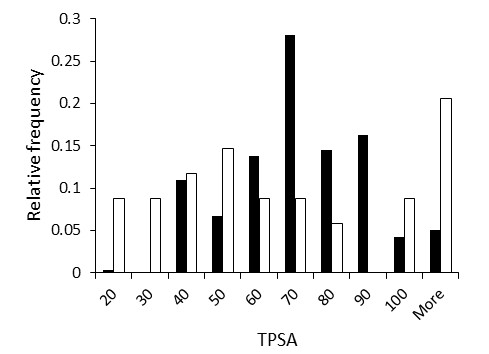


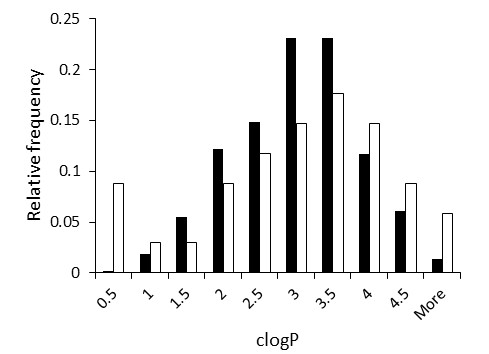

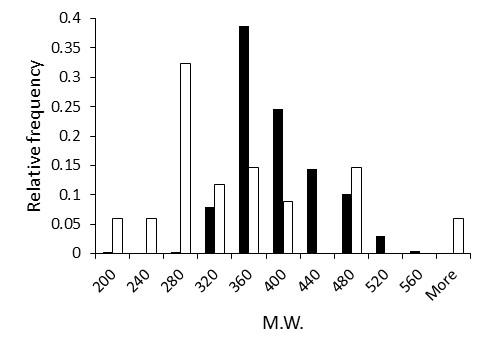


**Figure S5.** Distribution of log(K_p,uu,brain_) and physicochemical parameters, TPSA, clogP, and M.W. between internal training set (solid column) and the external test set (open column). TPSA, clogP and M.W. were calculated by StarDrop. The relative frequency was calculated by dividing a frequency count by sum of all frequencies. The number of compounds in training and test sets was 640 and 34, respectively.

**a b**

**
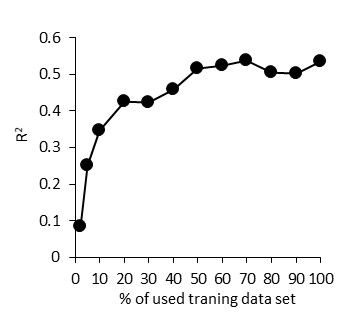

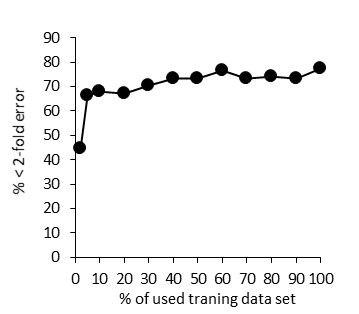
**

**Figure S6.** Impact of the number of compounds used for training on model performance using (a) R^2^, and (b) % compound within 2-fold error.

**Table S1. K_p,uu,brain_ Prediction Using RF and GPOPT with In Silico Efflux Ratio**

|  |  |  |  |  |  |
| --- | --- | --- | --- | --- | --- |
| Efflux ratio | | MDR1 | In silico | Not used | In silico |
|  |  | BCRP | Not used | In silico | In silico |
| RF | Cluster split | % < 2-fold | 68.0 | 68.0 | 68.8 |
|  |  | R^2^ | 0.355 | 0.352 | 0.371 |
|  |  | RMSE | 0.313 | 0.313 | 0.308 |
| GPOPT | Cluster split | % < 2-fold | 71.1 | 68.8 | 73.4 |
|  |  | R^2^ | 0.409 | 0.424 | 0.432 |
|  |  | RMSE | 0.308 | 0.301 | 0.304 |

*R^2^,* and RMSE were calculated using log(K_p,uu,brain_).

**Table S2. K_p,uu,brain_ Prediction Using the Neuropharmacokinetic Model with In Silico Efflux Ratio**

|  |  |  |
| --- | --- | --- |
|  | α [95% Cl] | 0.58 [0.44, 0.71] |
|  | β [95% Cl] | 1.2 [0.75, 1.6] |
| Cluster split | % < 2-fold | 55.5 |
|  | R^2^ | 0.268 |
|  | RMSE | 0.394 |

*R^2^,* and RMSE were calculated using log(K_p,uu,brain_).

**Table S3. Summary of External Dataset**

|  |  |  |  |  |  |
| --- | --- | --- | --- | --- | --- |
|  |  | Efflux ratio*^a^* | |  |  |
| Compound | M.W. | MDR1 | BCRP | K_p,uu,brain_ | Reference |
| Antipyrine | 188.2 | 0.91 | 0.96 | 0.857 | (17) |
| Bupropion | 239.7 | 1.2 | 0.80 | 2.00 | (11) |
| Carbamazepine | 236.3 | 1.26 | 0.88 | 0.389 | (30) |
| Citalopram | 324.4 | 18 | 0.60 | 0.623 | (30) |
| Daidzein | 254.2 | 0.30 | 4.1 | 0.0667 | (31) |
| Dantrolene | 313.2 | 1.3 | 48 | 0.0100 | (17) |
| Delavirdine | 456.6 | 110 | 38 | 0.043 | (11) |
| Diphenhydramine | 255.4 | 1.6 | 1.0 | 1.05 | (11) |
| Erlotinib | 393.4 | 19.8 | 5.8 | 0.0614 | (17) |
| Fluoxetine | 309.3 | 1.1 | 1.5 | 2.14 | (30) |
| Genistein | 270.2 | 0.19 | 26 | 0.0238 | (17) |
| Indomethacin | 357.8 | 4.4 | 20 | 0.11 | (11) |
| Lamotrigine | 256.1 | 1.44 | 1.2 | 1.40 | (30) |
| Loperamide | 477.0 | 92 | 0.97 | 0.0244 | (30) |
| Methotrexate | 454.4 | 1.42 | 1.2 | 0.006 | (11) |
| Metoprolol | 267.4 | 7.0 | 0.72 | 0.64 | (11) |
| Mirtazapine | 265.4 | 0.90 | 0.90 | 1.61 | (30) |
| Nelfinavir | 567.8 | 296 | 1.4 | 0.019 | (11) |
| Olanzapine | 312.4 | 2.0 | 1.2 | 2.45 | (30) |
| Pefloxacin | 333.4 | 9.9 | 17 | 0.199 | (31) |
| Phenytoin | 252.3 | 2.7 | 0.93 | 0.316 | (30) |
| Pindolol | 248.3 | 5.4 | 1.2 | 0.50 | (11) |
| Propranolol | 259.3 | 0.93 | 0.95 | 0.61 | (11) |
| Quinidine | 324.4 | 173 | 0.93 | 0.0359 | (17) |
| Rifampicin | 822.9 | 37 | 2.3 | 0.035 | (11) |
| Sertraline | 306.2 | 0.46 | 1.2 | 1.86 | (30) |
| Sorafenib | 464.8 | 4.8 | 33 | 0.0228 | (17) |
| Tacrine | 198.3 | 1.0 | 1.1 | 0.78 | (11) |
| Thioridazine | 370.6 | 2.6 | 0.57 | 1.43 | (30) |
| Topiramate | 339.4 | 25 | 0.70 | 0.33 | (11) |
| Trazodone | 371.9 | 0.63 | 0.89 | 0.957 | (30) |
| Venlafaxine | 277.4 | 7.6 | 0.70 | 1.07 | (30) |
| Verapamil | 454.6 | 23.1 | 0.84 | 0.053 | (11) |
| Zidovudine | 267.2 | 28 | 18 | 0.090 | (11) |

*^a^* Data cited from (17).

**Table S4. Summary of the Top 20 Molecular Descriptors of GPOPT on the Cluster and Time-Split Training Set**

|  |  |  |  |
| --- | --- | --- | --- |
| Molecular descriptor | Description | Molecular descriptor | Description |
| f004 | Number of para interactions between substituents singly or doubly bonded to the aromatic atoms | CH2link | Number of methylene-methylene groups |
| nHindole-like | Number of indole nitrogens with one hydrogen | UB | Number of triple, aromatic and double bonds. Bonds in nitro groups are not counted |
| ACamideO-nh-nh2 | Number of carboxamide groups with at least one hydrogen on the amide nitrogen | f443 | Number of methyl groups separated by two bonds from an aromatic atom |
| aaCH | Number of aromatic carbons with exactly one hydrogen | C2 | Number of sp3 carbons with no or exactly one hydrogen and connected to aliphatic carbons only |
| pyridine | Number of nitrogens in a pyridine ring | C21 | Number of aromatic carbons connected to two aromatic atoms and one aliphatic carbon |
| N2 | Number of uncharged aliphatic nitrogens with one hydrogen | nC(sp3) | Number of sp3 carbons |
| HBD-prof | Number of hydrogen bond donors including sulfur, oxygen and nitrogen atoms with higher specificity | f244 | Number of cyclic chains of four carbon atoms which have at least one hydrogen |
| H1a | Number of aliphatic carbons with one hydrogen | q481 | Number of aliphatic atoms in a ring with two singly bonded atoms connected and separated from an aromatic atom by four bonds |
| ACamideO-nh0 | Number of carboxamide groups with no hydrogen on the amide nitrogen | nNneutral | Number of neutral nitrogens |
| frg-8 | Number of para interactions in a disubstituted benzene ring with no other substituents | MW | Molecular weight |
| f440 | Number of methyl groups separated by three bonds from an aromatic atom | Vx | The McGowan volume |
| nCO | Number of carbonyl groups not in a carboxylic acid | q300 | Number of nitrogens in the alpha position of a branched substituent |
| p-hetero-or-halo | Number of heteroatoms (S,N,O and the first four halogens) para to heteroatoms (S,O,N and the first four halogens) | ERTLNoSPtPSA | Tha polar surface area for Nitrogen, Oxygen, Sulfur and Phosphorus atoms |

**Table S5. Summary of Internal Dataset**

|  |  |  |  |  |  |  |  |  |  |  |  |  |
| --- | --- | --- | --- | --- | --- | --- | --- | --- | --- | --- | --- | --- |
| Compound | Dataset | | Dose | Dosing | Collection time (h) | | ER | |  |  | K_p,brain_ | |
| No. | Cluster | Time | (mg/kg) | route | T1 | T2 | MDR1 | BCRP | f_u,plasma_ | f_u,brain_ | T1 | T2 |
| 1 | Test | Training | 1 | po | 1 | 4 | 9.6 | 0.8 | 0.020 | 0.006 | 1.125 | 1.142 |
| 2 | Training | Training | 3 | po | 0.5 | 1 | 1.5 | 2.0 | 0.300 | 0.119 | 1.333 | 1.283 |
| 3 | Test | Training | 1 | po | 1 | 2 | 10.7 | 1.5 | 0.050 | 0.019 | 0.610 | 0.486 |
| 4 | Training | Training | 3 | po | 1 | 4 | 1.9 | 1.4 | 0.110 | 0.047 | 0.392 | 0.621 |
| 5 | Test | Training | 0.2 | iv | 0.5 | 2 | 24.1 | 6.5 | 0.320 | 0.097 | 0.264 | 0.253 |
| 6 | Test | Training | 1 | po | 1 | 4 | 21.0 | 2.8 | 0.030 | 0.020 | 0.321 | 0.385 |
| 7 | Training | Training | 1 | po | 2 | 4 | 25.2 | 2.1 | 0.090 | 0.050 | 0.275 | 0.278 |
| 8 | Training | Training | 1 | po | 1 | 2 | 36.7 | 1.3 | 0.020 | 0.028 | 0.134 | 0.104 |
| 9 | Training | Training | 3 | po | 1 | 2 | 4.7 | 3.0 | 0.120 | 0.054 | 0.424 | 0.853 |
| 10 | Training | Training | 1 | po | 1 | 4 | 14.4 | 1.2 | 0.020 | 0.024 | 0.303 | 0.339 |
| 11 | Test | Training | 3 | po | 1 | 2 | 4.2 | 2.9 | 0.330 | 0.168 | 0.890 | 1.092 |
| 12 | Training | Training | 10 | sc | 0.5 | 2 | 16.3 | 2.2 | 0.240 | 0.133 | 0.256 | 0.213 |
| 13 | Training | Training | 3 | po | 0.5 | 1 | 2.0 | 3.3 | 0.140 | 0.043 | 0.899 | 0.769 |
| 14 | Training | Training | 1 | po | 2 | 4 | 34.1 | 1.9 | 0.030 | 0.019 | 0.240 | 0.249 |
| 15 | Test | Training | 1 | po | 2 | 4 | 8.0 | 1.0 | 0.030 | 0.017 | 0.413 | 0.410 |
| 16 | Training | Training | 10 | po | 1 | 2 | 2.4 | 37.6 | 0.060 | 0.007 | 1.737 | 1.501 |
| 17 | Training | Training | 3 | po | 1 | 2 | 8.0 | 3.9 | 0.290 | 0.116 | 0.483 | 0.453 |
| 18 | Training | Training | 1 | po | 1 | 1.5 | 29.2 | 5.0 | 0.120 | 0.030 | 0.493 | 0.469 |
| 19 | Training | Training | 0.2 | iv | 0.5 | 2 | 20.1 | 4.1 | 0.340 | 0.144 | 0.214 | 0.183 |
| 20 | Test | Training | 0.2 | iv | 0.5 | 1 | 29.1 | 5.2 | 0.330 | 0.157 | 0.259 | 0.202 |
| 21 | Test | Training | 1 | po | 2 | 4 | 5.7 | 0.9 | 0.030 | 0.014 | 0.939 | 0.907 |
| 22 | Training | Training | 1 | po | 2 | 4 | 40.8 | 2.5 | 0.020 | 0.024 | 0.179 | 0.224 |
| 23 | Training | Training | 1 | po | 1 | 2 | 45.8 | 1.2 | 0.020 | 0.020 | 0.140 | 0.137 |
| 24 | Test | Training | 3 | po | 1 | 2 | 2.6 | 2.9 | 0.230 | 0.102 | 0.450 | 0.351 |
| 25 | Training | Training | 3 | po | 0.5 | 2 | 1.5 | 1.6 | 0.230 | 0.096 | 0.479 | 0.726 |
| 26 | Training | Training | 1 | po | 1 | 2 | 59.5 | 9.5 | 0.050 | 0.028 | 0.054 | 0.058 |
| 27 | Training | Training | 0.2 | iv | 0.5 | 2 | 10.6 | 2.1 | 0.200 | 0.110 | 0.352 | 0.233 |
| 28 | Training | Training | 3 | po | 1 | 4 | 1.5 | 1.2 | 0.070 | 0.074 | 0.679 | 0.824 |
| 29 | Training | Training | 1 | po | 2 | 4 | 8.3 | 0.7 | 0.010 | 0.004 | 0.894 | 0.839 |
| 30 | Training | Training | 10 | sc | 0.5 | 2 | 37.1 | 1.3 | 0.540 | 0.280 | 0.141 | 0.094 |
| 31 | Training | Training | 1 | po | 1 | 4 | 41.4 | 23.3 | 0.210 | 0.107 | 1.057 | 0.767 |
| 32 | Training | Training | 3 | po | 1 | 2 | 0.9 | 0.8 | 0.050 | 0.023 | 0.844 | 0.719 |
| 33 | Training | Training | 1 | po | 1 | 2 | 8.7 | 1.2 | 0.030 | 0.010 | 1.261 | 1.203 |
| 34 | Training | Training | 3 | po | 1 | 4 | 17.3 | 5.1 | 0.200 | 0.064 | 0.364 | 0.462 |
| 35 | Training | Training | 1 | po | 4 | 8 | 33.3 | 3.0 | 0.020 | 0.009 | 0.157 | 0.139 |
| 36 | Training | Training | 30 | ip | 1 | 4 | 107.6 | 1.4 | 0.400 | 0.034 | 0.538 | 0.435 |
| 37 | Training | Training | 10 | sc | 0.5 | 2 | 54.7 | 1.0 | 0.350 | 0.290 | 0.092 | 0.090 |
| 38 | Training | Training | 10 | po | 1 | 2 | 2.3 | 1.0 | 0.760 | 0.429 | 1.931 | 2.194 |
| 39 | Training | Training | 3 | po | 1 | 2 | 26.2 | 1.5 | 0.040 | 0.022 | 0.083 | 0.257 |
| 40 | Training | Training | 3 | po | 1 | 2 | 1.2 | 1.2 | 0.010 | 0.010 | 0.742 | 0.617 |
| 41 | Training | Training | 1 | po | 1 | 4 | 22.0 | 1.1 | 0.010 | 0.022 | 0.227 | 0.242 |
| 42 | Test | Training | 3 | po | 1 | 4 | 18.9 | 6.9 | 0.160 | 0.096 | 0.365 | 0.215 |
| 43 | Training | Training | 1 | po | 1 | 2 | 7.6 | 1.1 | 0.070 | 0.032 | 0.635 | 0.682 |
| 44 | Training | Training | 1 | po | 0.5 | 1 | 10.0 | 2.2 | 0.060 | 0.021 | 0.795 | 0.922 |
| 45 | Test | Training | 10 | sc | 0.5 | 2 | 36.3 | 1.1 | 0.540 | 0.444 | 0.130 | 0.109 |
| 46 | Test | Training | 3 | po | 1 | 2 | 2.3 | 1.2 | 0.090 | 0.043 | 1.057 | 2.030 |
| 47 | Training | Training | 3 | po | 1 | 2 | 1.4 | 1.1 | 0.180 | 0.043 | 4.611 | 5.583 |
| 48 | Training | Training | 1 | po | 1 | 2 | 48.4 | 4.1 | 0.130 | 0.063 | 0.085 | 0.077 |
| 49 | Training | Training | 1 | po | 1 | 4 | 31.1 | 1.2 | 0.020 | 0.039 | 0.138 | 0.164 |
| 50 | Test | Training | 0.2 | iv | 0.5 | 1 | 31.0 | 12.8 | 0.330 | 0.126 | 0.580 | 0.488 |
| 51 | Training | Training | 3 | po | 0.5 | 1 | 5.4 | 4.0 | 0.290 | 0.112 | 0.422 | 0.364 |
| 52 | Training | Training | 10 | sc | 0.5 | 2 | 16.7 | 1.2 | 0.370 | 0.169 | 0.281 | 0.262 |
| 53 | Test | Training | 1 | po | 2 | 4 | 33.2 | 1.2 | 0.050 | 0.013 | 0.330 | 0.526 |
| 54 | Training | Training | 10 | po | 1 | 2 | 1.1 | 1.1 | 0.100 | 0.056 | 0.805 | 1.087 |
| 55 | Test | Training | 1 | po | 1 | 1.5 | 69.6 | 1.2 | 0.020 | 0.021 | 0.069 | 0.069 |
| 56 | Test | Training | 3 | po | 1 | 2 | 7.5 | 4.6 | 0.560 | 0.107 | 2.040 | 1.731 |
| 57 | Training | Training | 1 | po | 1 | 2 | 14.0 | 1.6 | 0.030 | 0.018 | 0.361 | 0.424 |
| 58 | Test | Training | 3 | po | 1 | 4 | 10.2 | 6.3 | 0.200 | 0.096 | 0.210 | 0.172 |
| 59 | Test | Training | 1 | po | 2 | 4 | 30.7 | 1.1 | 0.020 | 0.009 | 0.310 | 0.252 |
| 60 | Training | Training | 1 | po | 2 | 4 | 71.3 | 2.7 | 0.080 | 0.031 | 0.147 | 0.132 |
| 61 | Training | Training | 1 | po | 1 | 2 | 26.9 | 2.8 | 0.040 | 0.026 | 0.177 | 0.165 |
| 62 | Training | Training | 1 | po | 1 | 2 | 52.0 | 1.3 | 0.040 | 0.025 | 0.098 | 0.088 |
| 63 | Test | Training | 3 | po | 1 | 2 | 6.7 | 3.8 | 0.570 | 0.138 | 0.936 | 0.780 |
| 64 | Training | Training | 1 | po | 2 | 4 | 36.5 | 4.4 | 0.020 | 0.008 | 0.271 | 0.292 |
| 65 | Training | Training | 1 | po | 1 | 4 | 23.4 | 1.6 | 0.050 | 0.026 | 0.206 | 0.177 |
| 66 | Training | Training | 1 | po | 2 | 4 | 25.2 | 1.0 | 0.030 | 0.012 | 0.238 | 0.229 |
| 67 | Training | Training | 10 | iv | 1 | 3 | 2.1 | 0.7 | 0.100 | 0.033 | 2.669 | 2.751 |
| 68 | Training | Training | 1 | po | 1 | 4 | 7.9 | 1.6 | 0.070 | 0.018 | 4.388 | 2.778 |
| 69 | Training | Training | 3 | po | 1 | 4 | 1.3 | 1.1 | 0.010 | 0.003 | 2.585 | 2.601 |
| 70 | Training | Training | 1 | po | 1 | 4 | 17.8 | 1.2 | 0.050 | 0.036 | 0.353 | 0.426 |
| 71 | Training | Training | 1 | po | 2 | 4 | 28.0 | 2.8 | 0.030 | 0.010 | 0.313 | 0.322 |
| 72 | Training | Training | 3 | po | 1 | 2 | 9.3 | 5.4 | 0.330 | 0.092 | 0.498 | 0.515 |
| 73 | Training | Training | 1 | po | 2 | 4 | 24.0 | 1.5 | 0.120 | 0.032 | 0.366 | 0.382 |
| 74 | Training | Training | 1 | iv | 0.5 | 1 | 2.7 | 1.4 | 0.140 | 0.039 | 1.089 | 1.043 |
| 75 | Test | Test | 3 | po | 0.5 | 1 | 4.7 | 9.1 | 0.430 | 0.119 | 1.171 | 1.219 |
| 76 | Test | Test | 1 | iv | 0.083 | 0.25 | 48.0 | 4.1 | 0.090 | 0.041 | 0.290 | 0.139 |
| 77 | Test | Test | 3 | po | 0.5 | 1 | 146.4 | 10.1 | 0.320 | 0.166 | 0.076 | 0.072 |
| 78 | Test | Test | 1 | iv | 0.083 | 0.25 | 27.7 | 4.5 | 0.030 | 0.018 | 0.242 | 0.103 |
| 79 | Training | Training | 10 | po | 0.5 | 1 | 5.5 | 4.5 | 0.310 | 0.085 | 0.414 | 0.398 |
| 80 | Training | Test | 10 | po | 0.5 | 1 | 22.1 | 4.8 | 0.720 | 0.144 | 0.784 | 1.057 |
| 81 | Test | Training | 3 | po | 1 | 2 | 2.6 | 1.6 | 0.090 | 0.059 | 0.415 | 0.401 |
| 82 | Test | Training | 3 | po | 1 | 2 | 4.9 | 3.0 | 0.170 | 0.081 | 0.105 | 0.104 |
| 83 | Training | Training | 3 | po | 0.5 | 1 | 12.2 | 2.3 | 0.400 | 0.214 | 0.358 | 0.256 |
| 84 | Training | Training | 3 | po | 0.5 | 1 | 8.1 | 4.9 | 0.870 | 0.468 | 0.393 | 0.371 |
| 85 | Training | Training | 3 | po | 0.5 | 1 | 17.2 | 3.8 | 0.050 | 0.038 | 0.120 | 0.063 |
| 86 | Training | Training | 3 | po | 0.5 | 1 | 3.8 | 2.3 | 0.460 | 0.233 | 0.403 | 0.588 |
| 87 | Training | Training | 10 | ip | 0.5 | 0.75 | 6.7 | 1.7 | 0.450 | 0.111 | 2.459 | 1.940 |
| 88 | Test | Training | 3 | po | 1 | 2 | 2.8 | 1.4 | 0.030 | 0.025 | 0.223 | 0.207 |
| 89 | Test | Training | 3 | po | 1 | 2 | 6.8 | 2.2 | 0.240 | 0.143 | 0.283 | 0.268 |
| 90 | Training | Training | 3 | po | 0.5 | 1 | 8.1 | 3.4 | 0.340 | 0.200 | 0.144 | 0.101 |
| 91 | Training | Training | 3 | po | 0.5 | 1 | 9.4 | 5.8 | 0.660 | 0.248 | 0.303 | 0.362 |
| 92 | Training | Training | 1 | po | 0.5 | 1 | 61.2 | 64.4 | 0.400 | 0.103 | 0.000 | 0.031 |
| 93 | Training | Training | 10 | po | 0.75 | 1 | 7.2 | 3.4 | 0.180 | 0.104 | 0.193 | 0.216 |
| 94 | Training | Training | 3 | po | 0.5 | 1 | 0.9 | 0.8 | 0.180 | 0.080 | 0.704 | 1.005 |
| 95 | Test | Training | 3 | po | 1 | 2 | 4.8 | 4.2 | 0.310 | 0.110 | 0.182 | 0.222 |
| 96 | Training | Training | 3 | po | 1 | 2 | 8.0 | 3.1 | 0.110 | 0.069 | 0.131 | 0.138 |
| 97 | Test | Training | 3 | po | 0.5 | 1 | 10.3 | 4.3 | 0.100 | 0.074 | 0.220 | 0.185 |
| 98 | Training | Training | 3 | po | 0.5 | 1 | 4.7 | 3.1 | 0.080 | 0.060 | 0.104 | 0.102 |
| 99 | Training | Training | 3 | po | 0.5 | 1 | 46.9 | 12.3 | 0.180 | 0.178 | 0.036 | 0.052 |
| 100 | Test | Training | 10 | ip | 0.5 | 1 | 4.3 | 1.1 | 0.370 | 0.090 | 2.354 | 2.135 |
| 101 | Training | Training | 10 | ip | 0.5 | 0.75 | 44.3 | 0.7 | 0.620 | 0.216 | 0.361 | 0.301 |
| 102 | Training | Training | 3 | po | 1 | 2 | 4.6 | 3.9 | 0.500 | 0.213 | 0.310 | 0.353 |
| 103 | Training | Training | 3 | po | 1 | 2 | 8.5 | 3.3 | 0.140 | 0.085 | 0.099 | 0.093 |
| 104 | Training | Training | 3 | po | 0.5 | 1 | 21.2 | 3.5 | 0.560 | 0.268 | 0.174 | 0.161 |
| 105 | Training | Training | 3 | po | 0.5 | 1 | 1.8 | 1.2 | 0.230 | 0.057 | 1.132 | 0.943 |
| 106 | Training | Training | 3 | po | 0.5 | 1 | 0.8 | 1.0 | 0.030 | 0.019 | 0.775 | 0.834 |
| 107 | Training | Training | 3 | po | 0.5 | 1 | 56.8 | 11.6 | 0.090 | 0.153 | 0.014 | 0.011 |
| 108 | Training | Training | 10 | ip | 0.5 | 0.75 | 4.1 | 1.2 | 0.370 | 0.094 | 3.403 | 2.970 |
| 109 | Training | Training | 10 | ip | 0.5 | 0.75 | 1.9 | 0.9 | 0.160 | 0.027 | 1.326 | 2.035 |
| 110 | Training | Training | 3 | po | 0.5 | 1 | 3.2 | 2.7 | 0.260 | 0.168 | 0.321 | 0.274 |
| 111 | Training | Training | 3 | po | 1 | 2 | 2.9 | 1.9 | 0.070 | 0.025 | 0.580 | 0.554 |
| 112 | Training | Training | 3 | po | 0.5 | 1 | 3.7 | 6.8 | 0.510 | 0.064 | 0.346 | 0.326 |
| 113 | Training | Training | 3 | po | 0.5 | 1 | 4.5 | 3.0 | 0.450 | 0.198 | 0.423 | 0.391 |
| 114 | Training | Training | 3 | po | 0.5 | 1 | 1.9 | 1.5 | 0.230 | 0.116 | 0.938 | 0.966 |
| 115 | Training | Training | 3 | po | 0.5 | 1 | 3.3 | 2.3 | 0.150 | 0.050 | 0.280 | 0.251 |
| 116 | Training | Training | 2 | ip | 0.25 | 0.5 | 3.9 | 1.5 | 0.490 | 0.101 | 1.655 | 1.707 |
| 117 | Test | Training | 3 | po | 0.5 | 1 | 1.5 | 1.3 | 0.280 | 0.124 | 1.371 | 1.055 |
| 118 | Training | Training | 3 | po | 1 | 2 | 7.7 | 4.9 | 0.170 | 0.093 | 0.089 | 0.104 |
| 119 | Test | Training | 3 | po | 0.5 | 1 | 8.2 | 3.3 | 0.170 | 0.090 | 0.191 | 0.199 |
| 120 | Test | Training | 3 | po | 0.5 | 1 | 22.1 | 9.3 | 0.870 | 0.515 | 0.141 | 0.138 |
| 121 | Test | Training | 3 | po | 0.5 | 1 | 5.6 | 3.4 | 0.210 | 0.093 | 0.371 | 0.343 |
| 122 | Training | Training | 3 | po | 0.5 | 1 | 2.4 | 2.3 | 0.190 | 0.187 | 0.170 | 0.173 |
| 123 | Training | Training | 3 | po | 0.5 | 1 | 11.5 | 7.2 | 0.160 | 0.084 | 0.203 | 0.162 |
| 124 | Training | Training | 3 | po | 0.5 | 1 | 2.0 | 1.3 | 0.200 | 0.081 | 1.019 | 0.903 |
| 125 | Training | Training | 2 | ip | 0.5 | 0.75 | 2.5 | 1.2 | 0.110 | 0.016 | 2.547 | 2.080 |
| 126 | Training | Training | 3 | po | 0.5 | 1 | 1.7 | 1.2 | 0.200 | 0.056 | 0.908 | 0.995 |
| 127 | Training | Training | 3 | po | 0.5 | 1 | 16.9 | 10.1 | 0.270 | 0.312 | 0.040 | 0.043 |
| 128 | Training | Training | 3 | po | 0.5 | 1 | 1.4 | 2.1 | 0.160 | 0.052 | 1.255 | 0.952 |
| 129 | Training | Training | 3 | po | 0.5 | 1 | 14.4 | 3.7 | 0.290 | 0.068 | 1.544 | 1.231 |
| 130 | Training | Training | 3 | po | 0.5 | 1 | 2.8 | 2.8 | 0.320 | 0.195 | 0.487 | 0.378 |
| 131 | Test | Training | 3 | po | 0.5 | 1 | 15.2 | 1.3 | 0.350 | 0.134 | 0.177 | 0.143 |
| 132 | Training | Training | 3 | ip | 0.25 | 0.5 | 4.5 | 1.2 | 0.240 | 0.070 | 0.727 | 0.746 |
| 133 | Training | Training | 3 | po | 1 | 2 | 4.4 | 3.4 | 0.300 | 0.214 | 0.129 | 0.121 |
| 134 | Training | Training | 3 | po | 0.5 | 1 | 20.0 | 20.4 | 0.450 | 0.287 | 0.105 | 0.122 |
| 135 | Training | Training | 3 | po | 0.5 | 1 | 3.9 | 9.9 | 0.320 | 0.075 | 1.821 | 1.779 |
| 136 | Training | Training | 3 | po | 0.5 | 1 | 3.2 | 3.3 | 0.370 | 0.196 | 2.031 | 2.221 |
| 137 | Training | Training | 10 | po | 1 | 2 | 1.4 | 1.2 | 0.130 | 0.050 | 0.965 | 1.144 |
| 138 | Training | Training | 3 | po | 0.5 | 1 | 4.7 | 6.8 | 0.290 | 0.081 | 1.032 | 0.826 |
| 139 | Test | Training | 10 | ip | 0.5 | 0.75 | 5.4 | 2.3 | 0.210 | 0.060 | 0.364 | 0.330 |
| 140 | Test | Training | 10 | ip | 0.25 | 0.5 | 32.6 | 9.0 | 0.340 | 0.155 | 0.109 | 0.071 |
| 141 | Training | Training | 3 | po | 1 | 2 | 2.9 | 1.7 | 0.100 | 0.053 | 0.393 | 0.459 |
| 142 | Test | Training | 3 | po | 0.5 | 1 | 1.1 | 1.6 | 0.190 | 0.090 | 0.715 | 0.705 |
| 143 | Training | Training | 3 | po | 0.5 | 1 | 11.6 | 3.3 | 0.310 | 0.220 | 0.352 | 0.208 |
| 144 | Training | Training | 3 | po | 0.5 | 1 | 4.1 | 2.4 | 0.240 | 0.095 | 2.352 | 2.040 |
| 145 | Test | Training | 3 | po | 0.5 | 1 | 20.0 | 11.7 | 0.240 | 0.161 | 0.053 | 0.042 |
| 146 | Training | Training | 3 | po | 0.5 | 1 | 13.8 | 8.8 | 0.250 | 0.092 | 0.102 | 0.079 |
| 147 | Training | Training | 3 | ip | 0.5 | 0.75 | 14.3 | 3.1 | 0.480 | 0.232 | 0.697 | 0.482 |
| 148 | Training | Training | 3 | po | 1 | 2 | 1.9 | 1.4 | 0.210 | 0.144 | 0.535 | 0.420 |
| 149 | Training | Training | 3 | po | 0.5 | 1 | 4.8 | 4.7 | 0.770 | 0.434 | 0.499 | 0.464 |
| 150 | Training | Training | 3 | po | 0.5 | 1 | 14.4 | 4.4 | 0.600 | 0.278 | 0.196 | 0.149 |
| 151 | Training | Training | 10 | po | 0.5 | 1 | 2.4 | 1.0 | 0.550 | 0.303 | 0.493 | 0.428 |
| 152 | Training | Training | 3 | po | 0.5 | 1 | 14.7 | 8.6 | 0.120 | 0.159 | 0.027 | 0.043 |
| 153 | Training | Training | 3 | po | 0.5 | 1 | 11.9 | 11.0 | 0.220 | 0.055 | 0.291 | 0.277 |
| 154 | Training | Training | 1 | po | 2 | 4 | 4.9 | 1.8 | 0.020 | 0.003 | 2.370 | 1.913 |
| 155 | Training | Training | 3 | po | 0.5 | 1 | 29.8 | 16.2 | 0.120 | 0.042 | 0.154 | 0.184 |
| 156 | Training | Training | 3 | po | 0.5 | 1 | 5.3 | 4.3 | 0.270 | 0.106 | 0.764 | 0.858 |
| 157 | Training | Training | 3 | po | 0.5 | 1 | 31.5 | 7.3 | 0.400 | 0.186 | 0.219 | 0.220 |
| 158 | Training | Training | 30 | po | 0.5 | 1 | 15.1 | 2.7 | 0.290 | 0.116 | 0.232 | 0.173 |
| 159 | Training | Training | 10 | po | 0.5 | 1 | 8.9 | 6.5 | 0.180 | 0.127 | 0.460 | 0.422 |
| 160 | Training | Training | 3 | po | 0.5 | 1 | 0.8 | 1.0 | 0.040 | 0.019 | 0.409 | 0.508 |
| 161 | Training | Training | 3 | po | 0.5 | 1 | 5.4 | 4.1 | 0.140 | 0.046 | 1.381 | 1.357 |
| 162 | Training | Training | 10 | po | 0.5 | 1 | 11.4 | 7.8 | 0.440 | 0.246 | 0.371 | 0.301 |
| 163 | Training | Training | 10 | po | 0.5 | 1 | 4.4 | 4.7 | 0.330 | 0.131 | 0.758 | 0.589 |
| 164 | Training | Training | 3 | po | 0.5 | 1 | 6.8 | 6.4 | 0.400 | 0.128 | 0.716 | 0.542 |
| 165 | Training | Training | 3 | po | 0.5 | 1 | 1.7 | 2.0 | 0.240 | 0.094 | 0.852 | 0.904 |
| 166 | Training | Training | 10 | po | 0.5 | 1 | 14.2 | 8.7 | 0.420 | 0.168 | 0.284 | 0.328 |
| 167 | Training | Training | 3 | po | 0.5 | 1 | 0.9 | 1.3 | 0.010 | 0.002 | 1.003 | 1.314 |
| 168 | Training | Training | 10 | po | 0.5 | 1 | 5.9 | 3.3 | 0.070 | 0.048 | 0.911 | 0.849 |
| 169 | Training | Training | 3 | po | 0.5 | 1 | 1.9 | 1.5 | 0.050 | 0.016 | 1.006 | 1.068 |
| 170 | Training | Training | 1 | po | 0.5 | 1 | 7.1 | 6.2 | 0.160 | 0.076 | 0.460 | 0.173 |
| 171 | Training | Training | 3 | po | 0.5 | 1 | 4.0 | 2.2 | 0.100 | 0.036 | 0.706 | 0.718 |
| 172 | Training | Training | 3 | po | 0.5 | 1 | 2.1 | 1.5 | 0.090 | 0.035 | 0.706 | 0.457 |
| 173 | Test | Training | 10 | po | 0.5 | 1 | 7.2 | 10.9 | 0.500 | 0.238 | 0.153 | 0.123 |
| 174 | Training | Training | 3 | po | 0.5 | 1 | 75.7 | 9.4 | 0.520 | 0.098 | 0.353 | 0.358 |
| 175 | Training | Training | 3 | po | 0.5 | 1 | 3.6 | 1.7 | 0.110 | 0.121 | 0.239 | 0.310 |
| 176 | Training | Training | 10 | po | 0.5 | 1 | 5.5 | 2.3 | 0.140 | 0.041 | 1.116 | 1.344 |
| 177 | Training | Training | 3 | po | 0.5 | 1 | 1.6 | 1.9 | 0.280 | 0.070 | 0.509 | 0.797 |
| 178 | Training | Training | 3 | po | 0.5 | 1 | 8.1 | 5.4 | 0.310 | 0.159 | 1.427 | 1.702 |
| 179 | Training | Training | 10 | po | 0.5 | 1 | 19.9 | 9.6 | 0.450 | 0.202 | 0.280 | 0.283 |
| 180 | Training | Training | 3 | po | 0.5 | 1 | 4.6 | 2.2 | 0.040 | 0.055 | 0.503 | 0.367 |
| 181 | Training | Training | 3 | po | 0.5 | 1 | 13.2 | 2.4 | 0.330 | 0.069 | 0.917 | 1.588 |
| 182 | Training | Training | 3 | po | 0.5 | 1 | 87.4 | 14.5 | 0.210 | 0.040 | 0.100 | 0.077 |
| 183 | Training | Training | 1 | po | 0.5 | 1 | 11.7 | 3.6 | 0.110 | 0.049 | 0.286 | 0.281 |
| 184 | Training | Training | 1 | po | 0.5 | 1 | 92.6 | 100.3 | 0.180 | 0.073 | 0.031 | 0.037 |
| 185 | Test | Training | 3 | po | 0.5 | 1 | 14.9 | 4.4 | 0.420 | 0.133 | 1.011 | 1.496 |
| 186 | Training | Training | 3 | po | 0.5 | 1 | 14.8 | 6.9 | 0.180 | 0.118 | 0.447 | 0.373 |
| 187 | Training | Training | 10 | po | 0.5 | 1 | 15.0 | 8.3 | 0.410 | 0.197 | 0.338 | 0.290 |
| 188 | Training | Training | 1 | po | 0.5 | 1 | 8.3 | 2.7 | 0.090 | 0.036 | 0.245 | 0.292 |
| 189 | Training | Training | 3 | po | 0.5 | 1 | 21.2 | 3.7 | 0.360 | 0.070 | 1.161 | 2.051 |
| 190 | Training | Training | 3 | po | 0.5 | 1 | 31.6 | 4.8 | 0.180 | 0.023 | 0.378 | 0.434 |
| 191 | Training | Training | 3 | po | 0.5 | 1 | 1.4 | 1.1 | 0.100 | 0.031 | 0.480 | 0.390 |
| 192 | Training | Training | 3 | po | 0.5 | 1 | 10.1 | 2.4 | 0.210 | 0.069 | 0.554 | 0.488 |
| 193 | Training | Training | 3 | po | 0.5 | 1 | 5.0 | 3.9 | 0.230 | 0.069 | 0.272 | 0.188 |
| 194 | Training | Training | 3 | po | 0.5 | 1 | 12.0 | 5.7 | 0.230 | 0.106 | 0.567 | 0.385 |
| 195 | Training | Training | 3 | po | 0.5 | 1 | 18.9 | 2.2 | 0.350 | 0.198 | 0.167 | 0.219 |
| 196 | Training | Training | 3 | po | 0.5 | 1 | 6.8 | 2.6 | 0.050 | 0.074 | 0.137 | 0.115 |
| 197 | Test | Training | 3 | po | 0.5 | 1 | 17.4 | 1.8 | 0.300 | 0.034 | 0.446 | 0.225 |
| 198 | Training | Training | 3 | po | 0.5 | 1 | 5.0 | 5.4 | 0.290 | 0.119 | 0.919 | 0.836 |
| 199 | Test | Training | 1 | po | 0.5 | 1 | 14.0 | 4.5 | 0.180 | 0.090 | 0.304 | 0.245 |
| 200 | Training | Training | 3 | po | 0.5 | 1 | 2.8 | 2.6 | 0.390 | 0.085 | 0.694 | 0.620 |
| 201 | Training | Training | 10 | po | 0.5 | 1 | 6.3 | 2.3 | 0.070 | 0.012 | 0.480 | 0.601 |
| 202 | Training | Training | 30 | po | 0.5 | 1 | 4.1 | 1.2 | 0.170 | 0.054 | 0.694 | 0.655 |
| 203 | Training | Training | 3 | po | 0.5 | 1 | 15.3 | 3.6 | 0.290 | 0.130 | 0.256 | 0.346 |
| 204 | Test | Training | 3 | po | 0.5 | 1 | 3.9 | 3.0 | 0.300 | 0.099 | 0.913 | 0.720 |
| 205 | Training | Training | 3 | po | 0.5 | 1 | 105.7 | 45.9 | 0.530 | 0.148 | 0.063 | 0.180 |
| 206 | Training | Training | 1 | po | 0.5 | 1 | 10.1 | 4.8 | 0.060 | 0.027 | 0.320 | 0.433 |
| 207 | Test | Training | 3 | po | 0.5 | 1 | 1.0 | 1.2 | 0.020 | 0.014 | 1.603 | 1.197 |
| 208 | Training | Training | 3 | po | 0.5 | 1 | 2.0 | 2.3 | 0.460 | 0.104 | 1.352 | 1.165 |
| 209 | Training | Training | 3 | po | 0.5 | 1 | 27.8 | 6.1 | 0.460 | 0.194 | 0.284 | 0.250 |
| 210 | Training | Training | 10 | po | 0.5 | 1 | 15.3 | 2.5 | 0.390 | 0.213 | 0.177 | 0.196 |
| 211 | Training | Training | 3 | po | 0.5 | 1 | 7.1 | 1.9 | 0.240 | 0.181 | 0.185 | 0.196 |
| 212 | Training | Training | 3 | po | 0.5 | 1 | 45.3 | 2.9 | 0.600 | 0.100 | 0.215 | 0.193 |
| 213 | Test | Training | 3 | po | 0.5 | 1 | 65.6 | 40.9 | 0.270 | 0.062 | 0.000 | 0.200 |
| 214 | Training | Training | 1 | po | 0.5 | 1 | 3.5 | 3.8 | 0.470 | 0.103 | 1.076 | 1.084 |
| 215 | Training | Training | 3 | po | 0.5 | 1 | 1.3 | 1.2 | 0.220 | 0.037 | 2.657 | 3.384 |
| 216 | Training | Training | 3 | po | 0.5 | 1 | 2.5 | 3.9 | 0.140 | 0.039 | 0.714 | 0.751 |
| 217 | Training | Training | 10 | po | 0.5 | 1 | 29.0 | 4.1 | 0.260 | 0.084 | 0.239 | 0.267 |
| 218 | Training | Training | 3 | po | 0.5 | 1 | 1.1 | 1.1 | 0.010 | 0.003 | 1.179 | 0.877 |
| 219 | Training | Training | 3 | po | 0.5 | 1 | 12.2 | 6.3 | 0.510 | 0.194 | 0.500 | 0.561 |
| 220 | Training | Training | 3 | po | 0.5 | 1 | 69.4 | 10.3 | 0.210 | 0.038 | 0.141 | 0.161 |
| 221 | Training | Training | 1 | po | 0.5 | 1 | 15.5 | 4.6 | 0.090 | 0.034 | 0.293 | 0.183 |
| 222 | Training | Training | 1 | po | 0.5 | 1 | 1.6 | 1.2 | 0.320 | 0.089 | 1.451 | 1.236 |
| 223 | Training | Training | 3 | po | 0.5 | 1 | 2.1 | 1.9 | 0.100 | 0.067 | 0.592 | 0.287 |
| 224 | Training | Training | 3 | po | 0.5 | 1 | 7.2 | 2.7 | 0.030 | 0.021 | 0.534 | 0.670 |
| 225 | Training | Training | 1 | po | 0.5 | 1 | 8.6 | 2.2 | 0.280 | 0.091 | 0.591 | 0.587 |
| 226 | Training | Training | 3 | po | 0.5 | 1 | 15.3 | 6.6 | 0.230 | 0.081 | 0.618 | 0.504 |
| 227 | Test | Training | 3 | po | 0.5 | 1 | 33.7 | 12.1 | 0.360 | 0.116 | 0.341 | 0.301 |
| 228 | Training | Training | 3 | po | 0.5 | 1 | 1.0 | 1.0 | 0.080 | 0.055 | 0.352 | 0.282 |
| 229 | Training | Training | 3 | po | 0.5 | 1 | 7.9 | 5.2 | 0.210 | 0.061 | 1.624 | 1.399 |
| 230 | Training | Training | 3 | po | 0.5 | 1 | 22.6 | 3.4 | 0.420 | 0.243 | 0.208 | 0.171 |
| 231 | Training | Training | 3 | po | 0.5 | 1 | 1.2 | 2.2 | 0.060 | 0.018 | 0.407 | 0.393 |
| 232 | Training | Training | 3 | po | 1 | 2 | 10.2 | 5.2 | 0.480 | 0.158 | 0.495 | 0.468 |
| 233 | Training | Training | 1 | po | 0.5 | 1 | 18.8 | 19.5 | 0.090 | 0.033 | 0.134 | 0.108 |
| 234 | Training | Training | 3 | po | 0.5 | 1 | 28.2 | 12.9 | 0.360 | 0.118 | 0.184 | 0.154 |
| 235 | Test | Training | 3 | po | 1 | 2 | 1.4 | 1.3 | 0.190 | 0.089 | 0.199 | 0.077 |
| 236 | Training | Training | 3 | po | 0.5 | 1 | 1.0 | 0.9 | 0.170 | 0.040 | 1.170 | 1.515 |
| 237 | Training | Training | 3 | po | 0.5 | 1 | 2.2 | 1.2 | 0.190 | 0.079 | 0.805 | 1.956 |
| 238 | Training | Training | 1 | po | 0.5 | 1 | 1.0 | 1.1 | 0.090 | 0.056 | 0.683 | 0.489 |
| 239 | Training | Training | 1 | po | 0.5 | 1 | 4.2 | 2.5 | 0.050 | 0.018 | 0.658 | 0.445 |
| 240 | Training | Training | 3 | po | 1 | 2 | 12.7 | 4.5 | 0.430 | 0.124 | 0.387 | 0.259 |
| 241 | Training | Training | 1 | po | 0.5 | 1 | 20.1 | 5.3 | 0.100 | 0.117 | 0.213 | 0.158 |
| 242 | Training | Training | 3 | po | 0.5 | 1 | 3.6 | 2.9 | 0.440 | 0.191 | 0.345 | 0.364 |
| 243 | Training | Training | 3 | po | 1 | 2 | 3.1 | 3.2 | 0.380 | 0.153 | 0.265 | 0.274 |
| 244 | Training | Training | 1 | po | 0.5 | 1 | 64.3 | 10.6 | 0.190 | 0.245 | 0.000 | 0.017 |
| 245 | Training | Training | 3 | po | 0.5 | 1 | 1.1 | 1.0 | 0.290 | 0.102 | 1.399 | 1.555 |
| 246 | Training | Training | 1 | po | 0.5 | 1 | 50.0 | 3.3 | 0.080 | 0.071 | 0.095 | 0.073 |
| 247 | Training | Training | 3 | po | 1 | 2 | 5.9 | 4.4 | 0.300 | 0.134 | 0.358 | 0.350 |
| 248 | Test | Training | 3 | po | 0.5 | 1 | 8.6 | 4.4 | 0.320 | 0.131 | 0.334 | 0.264 |
| 249 | Training | Training | 30 | po | 0.5 | 1 | 107.2 | 1.0 | 0.380 | 0.023 | 0.513 | 0.846 |
| 250 | Training | Training | 3 | po | 0.5 | 1 | 85.1 | 41.3 | 0.600 | 0.270 | 0.018 | 0.016 |
| 251 | Training | Training | 3 | po | 0.5 | 1 | 2.5 | 1.7 | 0.310 | 0.122 | 1.224 | 1.542 |
| 252 | Training | Training | 1 | po | 0.5 | 1 | 10.8 | 2.4 | 0.010 | 0.053 | 0.063 | 0.106 |
| 253 | Training | Training | 3 | po | 0.5 | 1 | 19.8 | 7.2 | 0.310 | 0.110 | 0.169 | 0.149 |
| 254 | Training | Training | 1 | po | 0.5 | 1 | 45.8 | 4.6 | 0.040 | 0.068 | 0.037 | 0.051 |
| 255 | Training | Training | 3 | po | 1 | 2 | 10.8 | 4.9 | 0.370 | 0.128 | 0.317 | 0.458 |
| 256 | Training | Training | 3 | po | 1 | 2 | 13.4 | 3.5 | 0.350 | 0.126 | 0.187 | 0.289 |
| 257 | Training | Training | 3 | po | 1 | 2 | 60.1 | 26.2 | 0.520 | 0.213 | 0.083 | 0.060 |
| 258 | Training | Training | 3 | po | 1 | 2 | 2.7 | 1.7 | 0.240 | 0.222 | 0.167 | 0.278 |
| 259 | Training | Training | 3 | po | 0.5 | 1 | 1.9 | 2.4 | 0.230 | 0.066 | 1.278 | 3.386 |
| 260 | Training | Training | 3 | po | 1 | 2 | 6.0 | 2.3 | 0.390 | 0.193 | 0.331 | 0.287 |
| 261 | Test | Training | 3 | po | 1 | 2 | 1.5 | 1.6 | 0.120 | 0.032 | 0.711 | 0.700 |
| 262 | Training | Training | 1 | po | 0.5 | 1 | 3.4 | 5.8 | 0.050 | 0.026 | 0.280 | 0.333 |
| 263 | Training | Training | 1 | po | 0.5 | 1 | 1.9 | 1.6 | 0.040 | 0.009 | 1.051 | 0.753 |
| 264 | Test | Training | 3 | po | 0.5 | 1 | 33.6 | 11.8 | 0.720 | 0.170 | 0.213 | 0.210 |
| 265 | Training | Training | 3 | po | 1 | 2 | 3.5 | 3.5 | 0.080 | 0.026 | 0.750 | 0.614 |
| 266 | Training | Training | 3 | po | 0.5 | 1 | 97.9 | 44.9 | 0.620 | 0.275 | 0.037 | 0.037 |
| 267 | Training | Training | 10 | po | 0.5 | 1 | 1.1 | 0.9 | 0.230 | 0.162 | 0.707 | 0.792 |
| 268 | Test | Training | 3 | po | 0.5 | 1 | 1.0 | 1.5 | 0.160 | 0.049 | 0.472 | 0.383 |
| 269 | Training | Training | 3 | po | 0.5 | 1 | 21.5 | 6.8 | 0.420 | 0.227 | 0.054 | 0.044 |
| 270 | Training | Training | 1 | po | 0.5 | 1 | 10.7 | 30.4 | 0.080 | 0.042 | 0.049 | 0.018 |
| 271 | Training | Training | 1 | po | 0.5 | 1 | 1.0 | 1.3 | 0.010 | 0.003 | 0.732 | 0.606 |
| 272 | Training | Training | 2 | po | 1 | 2 | 12.0 | 6.3 | 0.030 | 0.014 | 0.593 | 0.680 |
| 273 | Test | Training | 3 | po | 0.5 | 1 | 1.1 | 0.9 | 0.140 | 0.050 | 2.201 | 1.837 |
| 274 | Training | Training | 3 | po | 2 | 4 | 3.5 | 0.7 | 0.010 | 0.004 | 2.948 | 2.655 |
| 275 | Training | Training | 3 | po | 1 | 2 | 22.6 | 3.4 | 0.330 | 0.197 | 0.109 | 0.110 |
| 276 | Training | Training | 3 | po | 1 | 2 | 4.5 | 3.1 | 0.080 | 0.051 | 0.184 | 0.173 |
| 277 | Training | Training | 3 | po | 0.5 | 1 | 3.6 | 5.5 | 0.400 | 0.144 | 0.229 | 0.263 |
| 278 | Test | Training | 3 | po | 1 | 2 | 13.1 | 8.5 | 0.440 | 0.110 | 1.163 | 0.974 |
| 279 | Training | Training | 1 | po | 0.5 | 1 | 3.4 | 4.2 | 0.010 | 0.004 | 0.430 | 0.531 |
| 280 | Training | Training | 3 | po | 1 | 2 | 60.3 | 19.5 | 0.610 | 0.175 | 0.081 | 0.107 |
| 281 | Training | Training | 3 | po | 0.5 | 1 | 1.6 | 1.2 | 0.200 | 0.064 | 1.893 | 1.982 |
| 282 | Test | Training | 3 | po | 0.5 | 1 | 2.4 | 1.3 | 0.310 | 0.170 | 0.769 | 0.809 |
| 283 | Training | Training | 3 | po | 1 | 2 | 15.0 | 83.2 | 0.220 | 0.070 | 0.066 | 0.071 |
| 284 | Training | Training | 3 | ip | 0.5 | 1 | 2.0 | 1.6 | 0.050 | 0.036 | 0.483 | 0.346 |
| 285 | Training | Training | 3 | po | 0.5 | 1 | 25.3 | 32.7 | 0.350 | 0.102 | 0.110 | 0.077 |
| 286 | Training | Training | 3 | po | 1 | 2 | 9.5 | 3.0 | 0.380 | 0.140 | 0.332 | 0.412 |
| 287 | Training | Training | 3 | po | 1 | 2 | 2.1 | 1.2 | 0.070 | 0.012 | 2.576 | 1.928 |
| 288 | Training | Training | 10 | ip | 0.5 | 2 | 40.5 | 1.1 | 0.840 | 0.152 | 0.685 | 1.681 |
| 289 | Training | Training | 3 | po | 1 | 2 | 26.6 | 25.4 | 0.480 | 0.218 | 0.088 | 0.048 |
| 290 | Training | Training | 3 | po | 1 | 2 | 1.5 | 0.7 | 0.150 | 0.054 | 1.097 | 0.965 |
| 291 | Test | Training | 3 | po | 1 | 2 | 1.9 | 1.3 | 0.180 | 0.075 | 0.549 | 0.490 |
| 292 | Training | Training | 3 | po | 0.5 | 1 | 8.6 | 4.1 | 0.540 | 0.152 | 0.198 | 0.256 |
| 293 | Training | Training | 3 | po | 0.5 | 1 | 37.0 | 73.7 | 0.250 | 0.072 | 0.153 | 0.146 |
| 294 | Training | Training | 3 | po | 1 | 2 | 5.0 | 3.0 | 0.310 | 0.100 | 0.730 | 0.646 |
| 295 | Training | Training | 3 | po | 1 | 2 | 6.3 | 4.6 | 0.350 | 0.130 | 0.540 | 0.546 |
| 296 | Training | Training | 3 | po | 1 | 2 | 9.0 | 6.9 | 0.540 | 0.208 | 0.116 | 0.174 |
| 297 | Test | Training | 3 | po | 1 | 2 | 18.9 | 5.7 | 0.290 | 0.108 | 0.445 | 0.279 |
| 298 | Training | Training | 3 | po | 1 | 2 | 3.8 | 3.6 | 0.180 | 0.138 | 0.152 | 0.135 |
| 299 | Training | Training | 3 | po | 1 | 2 | 8.5 | 4.6 | 0.150 | 0.116 | 0.101 | 0.106 |
| 300 | Training | Training | 3 | po | 0.5 | 1 | 17.0 | 9.2 | 0.460 | 0.100 | 0.845 | 0.584 |
| 301 | Training | Training | 3 | po | 1 | 2 | 2.6 | 1.8 | 0.120 | 0.041 | 0.628 | 0.524 |
| 302 | Test | Training | 3 | po | 1 | 2 | 25.1 | 23.4 | 0.370 | 0.102 | 0.092 | 0.160 |
| 303 | Training | Training | 3 | po | 1 | 2 | 3.6 | 2.5 | 0.270 | 0.075 | 0.908 | 0.739 |
| 304 | Training | Training | 3 | po | 0.5 | 1 | 28.3 | 17.7 | 0.490 | 0.158 | 0.103 | 0.101 |
| 305 | Training | Training | 3 | po | 0.5 | 1 | 10.1 | 4.8 | 0.500 | 0.165 | 0.312 | 0.243 |
| 306 | Training | Training | 3 | po | 0.5 | 1 | 1.7 | 1.3 | 0.290 | 0.121 | 0.738 | 0.772 |
| 307 | Training | Training | 3 | po | 0.5 | 1 | 1.1 | 1.2 | 0.220 | 0.099 | 1.941 | 2.049 |
| 308 | Training | Training | 3 | po | 1 | 2 | 3.5 | 3.8 | 0.450 | 0.146 | 0.149 | 0.411 |
| 309 | Training | Test | 1 | iv | 0.083 | 0.25 | 2.8 | 0.6 | 0.020 | 0.011 | 1.503 | 0.960 |
| 310 | Training | Test | 1 | iv | 0.083 | 0.25 | 8.3 | 1.8 | 0.030 | 0.012 | 0.658 | 0.346 |
| 311 | Training | Test | 3 | po | 0.5 | 1 | 1.6 | 2.0 | 0.610 | 0.285 | 1.092 | 0.863 |
| 312 | Training | Test | 1 | iv | 0.083 | 0.25 | 8.6 | 2.0 | 0.050 | 0.025 | 0.831 | 0.494 |
| 313 | Training | Test | 1 | po | 0.5 | 1 | 3.5 | 1.0 | 0.010 | 0.004 | 0.716 | 0.649 |
| 314 | Test | Test | 3 | po | 0.5 | 1 | 1.6 | 1.3 | 0.240 | 0.112 | 0.468 | 0.418 |
| 315 | Training | Test | 3 | po | 0.5 | 1 | 1.7 | 1.2 | 0.110 | 0.036 | 1.041 | 1.360 |
| 316 | Training | Test | 1 | iv | 0.083 | 0.25 | 5.3 | 1.6 | 0.020 | 0.003 | 3.950 | 3.271 |
| 317 | Training | Test | 1 | iv | 0.083 | 0.25 | 1.7 | 0.8 | 0.100 | 0.040 | 2.163 | 1.594 |
| 318 | Training | Test | 3 | po | 0.5 | 1 | 2.6 | 0.9 | 0.390 | 0.250 | 0.150 | 0.235 |
| 319 | Training | Test | 1 | iv | 0.083 | 0.25 | 8.1 | 1.4 | 0.040 | 0.053 | 0.263 | 0.192 |
| 320 | Training | Test | 1 | iv | 0.083 | 0.25 | 43.3 | 5.1 | 0.060 | 0.024 | 0.452 | 0.185 |
| 321 | Training | Test | 1 | iv | 0.083 | 0.25 | 2.1 | 0.7 | 0.020 | 0.005 | 3.095 | 2.230 |
| 322 | Test | Test | 3 | po | 0.5 | 1 | 1.8 | 1.3 | 0.350 | 0.152 | 1.000 | 0.859 |
| 323 | Test | Test | 10 | po | 0.5 | 1 | 2.8 | 2.1 | 0.340 | 0.082 | 1.391 | 1.543 |
| 324 | Test | Test | 1 | iv | 0.083 | 0.25 | 4.4 | 1.4 | 0.010 | 0.002 | 4.081 | 6.447 |
| 325 | Training | Training | 1 | po | 1 | 4 | 6.2 | 1.2 | 0.080 | 0.022 | 1.407 | 1.885 |
| 326 | Training | Training | 3 | po | 0.5 | 1 | 4.0 | 2.6 | 0.350 | 0.170 | 0.766 | 0.756 |
| 327 | Test | Training | 3 | po | 1 | 2 | 10.5 | 1.3 | 0.140 | 0.064 | 0.393 | 0.386 |
| 328 | Training | Training | 3 | po | 1 | 2 | 5.7 | 3.5 | 0.160 | 0.062 | 0.449 | 0.520 |
| 329 | Training | Training | 3 | ip | 0.5 | 1 | 5.9 | 1.8 | 0.080 | 0.020 | 0.455 | 0.572 |
| 330 | Training | Training | 3 | ip | 0.5 | 1 | 3.6 | 1.5 | 0.030 | 0.008 | 1.062 | 1.278 |
| 331 | Test | Training | 3 | po | 1 | 2 | 8.8 | 5.0 | 0.590 | 0.205 | 0.506 | 0.471 |
| 332 | Training | Training | 3 | po | 0.5 | 1 | 19.2 | 19.5 | 0.260 | 0.068 | 0.747 | 0.820 |
| 333 | Training | Training | 3 | po | 1 | 2 | 1.5 | 1.0 | 0.150 | 0.041 | 7.034 | 4.357 |
| 334 | Test | Training | 3 | po | 0.5 | 1 | 8.4 | 5.1 | 0.490 | 0.137 | 0.847 | 0.791 |
| 335 | Training | Training | 3 | po | 1 | 2 | 42.2 | 4.9 | 0.160 | 0.046 | 0.442 | 0.399 |
| 336 | Training | Training | 3 | po | 1 | 2 | 5.7 | 4.7 | 0.240 | 0.066 | 0.561 | 0.570 |
| 337 | Training | Training | 3 | po | 0.5 | 1 | 22.8 | 19.6 | 0.300 | 0.086 | 0.197 | 0.181 |
| 338 | Test | Training | 3 | ip | 0.5 | 1 | 3.8 | 1.9 | 0.010 | 0.005 | 0.210 | 0.216 |
| 339 | Training | Training | 3 | po | 1 | 2 | 6.5 | 2.2 | 0.320 | 0.116 | 0.629 | 0.744 |
| 340 | Test | Training | 3 | po | 0.5 | 1 | 3.0 | 2.5 | 0.270 | 0.044 | 0.603 | 1.370 |
| 341 | Training | Training | 3 | po | 0.5 | 1 | 3.7 | 0.9 | 0.080 | 0.021 | 1.384 | 1.559 |
| 342 | Training | Training | 3 | po | 0.5 | 1 | 1.1 | 0.9 | 0.120 | 0.127 | 1.481 | 2.667 |
| 343 | Test | Training | 3 | po | 1 | 2 | 32.6 | 2.1 | 0.280 | 0.105 | 0.109 | 0.128 |
| 344 | Training | Training | 3 | po | 0.5 | 1 | 24.5 | 1.8 | 0.100 | 0.029 | 0.317 | 0.322 |
| 345 | Training | Training | 3 | po | 0.5 | 1 | 20.3 | 1.8 | 0.100 | 0.046 | 0.119 | 0.127 |
| 346 | Training | Training | 3 | po | 1 | 2 | 20.5 | 8.0 | 0.710 | 0.233 | 0.264 | 0.309 |
| 347 | Test | Training | 3 | po | 0.5 | 1 | 13.6 | 5.5 | 0.240 | 0.064 | 0.238 | 0.195 |
| 348 | Training | Training | 3 | po | 1 | 2 | 5.7 | 1.8 | 0.450 | 0.151 | 0.746 | 0.782 |
| 349 | Training | Training | 3 | po | 0.5 | 1 | 2.7 | 1.6 | 0.230 | 0.094 | 1.552 | 1.318 |
| 350 | Test | Training | 3 | po | 1 | 2 | 23.0 | 3.5 | 0.110 | 0.038 | 0.200 | 0.198 |
| 351 | Training | Training | 3 | po | 1 | 2 | 3.2 | 9.2 | 0.110 | 0.052 | 0.659 | 0.472 |
| 352 | Training | Training | 3 | po | 0.5 | 1 | 6.0 | 3.7 | 0.470 | 0.121 | 0.670 | 0.698 |
| 353 | Test | Training | 3 | po | 1 | 2 | 5.6 | 1.1 | 0.210 | 0.059 | 0.606 | 0.603 |
| 354 | Test | Training | 3 | po | 0.5 | 1 | 3.2 | 2.0 | 0.620 | 0.207 | 0.827 | 0.367 |
| 355 | Training | Training | 3 | po | 0.5 | 1 | 20.0 | 2.0 | 0.470 | 0.214 | 0.102 | 0.103 |
| 356 | Training | Training | 3 | po | 1 | 2 | 7.3 | 5.1 | 0.510 | 0.098 | 1.400 | 1.377 |
| 357 | Test | Training | 3 | po | 0.5 | 1 | 2.7 | 1.6 | 0.190 | 0.049 | 2.291 | 2.882 |
| 358 | Training | Training | 3 | po | 1 | 2 | 31.7 | 1.8 | 0.170 | 0.064 | 0.255 | 0.180 |
| 359 | Training | Training | 3 | po | 1 | 2 | 4.6 | 11.7 | 0.030 | 0.016 | 0.030 | 0.027 |
| 360 | Training | Training | 3 | po | 1 | 2 | 24.1 | 2.4 | 0.150 | 0.085 | 0.073 | 0.078 |
| 361 | Training | Training | 3 | po | 1 | 2 | 12.0 | 10.8 | 0.180 | 0.041 | 0.195 | 0.177 |
| 362 | Training | Training | 3 | po | 0.5 | 1 | 2.8 | 1.4 | 0.110 | 0.043 | 0.926 | 0.970 |
| 363 | Training | Training | 10 | po | 0.5 | 1 | 1.2 | 0.8 | 0.070 | 0.043 | 0.360 | 0.360 |
| 364 | Training | Training | 3 | po | 1 | 2 | 2.3 | 1.2 | 0.280 | 0.075 | 3.958 | 4.683 |
| 365 | Training | Training | 3 | po | 0.5 | 1 | 3.7 | 1.4 | 0.210 | 0.050 | 2.206 | 1.629 |
| 366 | Training | Training | 3 | po | 1 | 2 | 45.1 | 28.5 | 0.440 | 0.153 | 0.128 | 0.168 |
| 367 | Training | Training | 3 | po | 0.5 | 1 | 12.0 | 1.4 | 0.150 | 0.071 | 0.125 | 0.134 |
| 368 | Training | Training | 3 | po | 0.5 | 1 | 14.0 | 6.0 | 0.230 | 0.064 | 0.155 | 0.096 |
| 369 | Training | Training | 3 | ip | 0.5 | 1 | 1.1 | 0.7 | 0.020 | 0.005 | 1.640 | 0.500 |
| 370 | Training | Training | 3 | po | 0.5 | 1 | 5.7 | 2.3 | 0.290 | 0.099 | 0.474 | 0.518 |
| 371 | Training | Training | 3 | po | 0.5 | 1 | 2.8 | 1.7 | 0.260 | 0.071 | 3.188 | 3.024 |
| 372 | Training | Training | 3 | po | 0.5 | 1 | 1.3 | 1.0 | 0.150 | 0.043 | 1.820 | 1.358 |
| 373 | Training | Training | 3 | po | 1 | 2 | 8.7 | 1.1 | 0.080 | 0.025 | 1.090 | 0.810 |
| 374 | Training | Training | 1 | po | 0.5 | 1 | 13.7 | 4.3 | 0.160 | 0.044 | 0.166 | 0.166 |
| 375 | Training | Training | 3 | po | 0.5 | 1 | 6.4 | 2.6 | 0.060 | 0.018 | 0.381 | 0.354 |
| 376 | Training | Training | 3 | po | 1 | 2 | 13.2 | 2.3 | 0.150 | 0.111 | 0.121 | 0.145 |
| 377 | Training | Training | 3 | po | 1 | 2 | 6.5 | 1.1 | 0.100 | 0.039 | 0.330 | 0.219 |
| 378 | Test | Training | 3 | po | 0.5 | 1 | 2.2 | 0.9 | 0.270 | 0.093 | 0.865 | 0.814 |
| 379 | Training | Training | 3 | po | 0.5 | 1 | 1.0 | 1.0 | 0.240 | 0.136 | 1.501 | 1.655 |
| 380 | Training | Training | 1 | po | 1 | 2 | 17.0 | 1.4 | 0.120 | 0.038 | 0.383 | 0.413 |
| 381 | Training | Training | 1 | po | 0.5 | 1 | 8.6 | 7.7 | 0.310 | 0.076 | 0.152 | 0.131 |
| 382 | Test | Training | 3 | po | 0.5 | 1 | 32.6 | 6.2 | 0.220 | 0.077 | 0.090 | 0.074 |
| 383 | Training | Training | 3 | iv | 0.083 | 0.25 | 4.5 | 1.1 | 0.490 | 0.058 | 7.852 | 10.767 |
| 384 | Training | Training | 3 | po | 0.5 | 1 | 6.1 | 1.3 | 0.140 | 0.034 | 0.550 | 0.576 |
| 385 | Test | Training | 3 | po | 0.5 | 1 | 4.7 | 2.2 | 0.400 | 0.172 | 0.970 | 0.910 |
| 386 | Test | Training | 3 | po | 0.5 | 1 | 1.1 | 1.3 | 0.090 | 0.051 | 0.990 | 1.133 |
| 387 | Training | Training | 3 | po | 1 | 2 | 22.7 | 6.0 | 0.160 | 0.084 | 0.217 | 0.215 |
| 388 | Training | Training | 3 | po | 1 | 2 | 1.3 | 2.2 | 0.030 | 0.010 | 0.949 | 0.979 |
| 389 | Training | Training | 3 | po | 1 | 2 | 7.3 | 1.7 | 0.140 | 0.056 | 0.233 | 0.219 |
| 390 | Training | Training | 3 | po | 0.5 | 1 | 18.7 | 4.9 | 0.080 | 0.019 | 0.352 | 0.363 |
| 391 | Training | Training | 3 | po | 0.5 | 1 | 9.5 | 8.0 | 0.510 | 0.123 | 2.741 | 1.967 |
| 392 | Training | Training | 10 | po | 0.5 | 1 | 7.9 | 1.8 | 0.180 | 0.090 | 0.199 | 0.155 |
| 393 | Training | Training | 3 | po | 0.5 | 1 | 1.0 | 1.8 | 0.110 | 0.037 | 2.437 | 2.808 |
| 394 | Test | Training | 3 | po | 1 | 2 | 4.4 | 8.6 | 0.530 | 0.261 | 0.606 | 0.780 |
| 395 | Training | Training | 3 | po | 1 | 2 | 2.6 | 6.6 | 0.390 | 0.101 | 0.975 | 0.804 |
| 396 | Test | Training | 3 | po | 1 | 2 | 2.5 | 19.2 | 0.040 | 0.014 | 0.388 | 0.466 |
| 397 | Training | Training | 3 | po | 1 | 2 | 0.8 | 1.1 | 0.180 | 0.078 | 0.587 | 0.459 |
| 398 | Test | Training | 3 | po | 0.5 | 1 | 52.4 | 5.3 | 0.170 | 0.052 | 0.164 | 0.187 |
| 399 | Training | Training | 3 | po | 0.5 | 1 | 2.3 | 2.9 | 0.250 | 0.069 | 1.168 | 1.165 |
| 400 | Test | Training | 3 | po | 0.5 | 1 | 5.4 | 1.2 | 0.130 | 0.032 | 0.810 | 0.717 |
| 401 | Training | Test | 3 | po | 0.5 | 1 | 2.9 | 1.0 | 0.270 | 0.228 | 0.180 | 0.160 |
| 402 | Training | Test | 1 | po | 0.5 | 1 | 6.8 | 1.2 | 0.030 | 0.011 | 0.544 | 0.650 |
| 403 | Training | Test | 3 | po | 0.5 | 1 | 4.4 | 1.6 | 0.040 | 0.020 | 0.347 | 0.498 |
| 404 | Training | Test | 1 | iv | 0.083 | 0.25 | 36.7 | 2.4 | 0.060 | 0.061 | 0.086 | 0.036 |
| 405 | Training | Test | 1 | po | 0.5 | 1 | 3.5 | 1.5 | 0.010 | 0.005 | 0.482 | 0.584 |
| 406 | Test | Test | 3 | po | 0.5 | 1 | 8.6 | 1.5 | 0.140 | 0.052 | 0.483 | 0.638 |
| 407 | Test | Test | 1 | iv | 0.083 | 0.25 | 3.9 | 1.1 | 0.020 | 0.005 | 0.965 | 0.742 |
| 408 | Test | Test | 1 | iv | 0.083 | 0.25 | 13.7 | 1.1 | 0.030 | 0.038 | 0.580 | 0.073 |
| 409 | Training | Test | 1 | iv | 0.083 | 0.25 | 5.3 | 1.3 | 0.020 | 0.023 | 0.222 | 0.164 |
| 410 | Training | Test | 3 | po | 0.5 | 1 | 10.3 | 2.7 | 0.030 | 0.010 | 0.281 | 0.277 |
| 411 | Training | Test | 3 | po | 0.5 | 1 | 1.4 | 2.2 | 0.460 | 0.234 | 0.589 | 0.646 |
| 412 | Training | Test | 3 | po | 0.5 | 1 | 4.4 | 4.7 | 0.190 | 0.101 | 0.400 | 0.434 |
| 413 | Training | Test | 3 | po | 0.5 | 1 | 3.5 | 1.3 | 0.020 | 0.014 | 0.564 | 0.624 |
| 414 | Training | Test | 1 | po | 0.5 | 1 | 12.8 | 3.8 | 0.160 | 0.080 | 0.303 | 0.300 |
| 415 | Test | Test | 10 | po | 0.5 | 1 | 13.9 | 6.7 | 0.640 | 0.206 | 1.143 | 1.386 |
| 416 | Training | Test | 3 | po | 0.5 | 1 | 2.7 | 1.8 | 0.120 | 0.048 | 1.170 | 1.091 |
| 417 | Training | Training | 3 | po | 0.5 | 1 | 31.9 | 22.0 | 0.360 | 0.133 | 0.235 | 0.251 |
| 418 | Training | Training | 10 | po | 0.5 | 1 | 3.6 | 5.3 | 0.320 | 0.112 | 0.677 | 0.622 |
| 419 | Training | Training | 1 | po | 0.5 | 1 | 0.7 | 1.0 | 0.220 | 0.077 | 1.849 | 1.895 |
| 420 | Test | Training | 1 | po | 0.5 | 1 | 13.7 | 7.4 | 0.160 | 0.072 | 0.252 | 0.271 |
| 421 | Training | Test | 3 | po | 0.5 | 1 | 2.0 | 2.8 | 0.360 | 0.145 | 0.885 | 1.170 |
| 422 | Training | Training | 1 | po | 2 | 4 | 23.5 | 1.1 | 0.030 | 0.010 | 0.224 | 0.270 |
| 423 | Training | Training | 10 | po | 1 | 4 | 6.0 | 5.1 | 0.030 | 0.006 | 0.490 | 0.536 |
| 424 | Training | Training | 3 | po | 0.5 | 1 | 26.3 | 24.7 | 0.400 | 0.078 | 0.109 | 0.131 |
| 425 | Training | Training | 10 | po | 0.5 | 1 | 4.4 | 6.3 | 0.330 | 0.112 | 0.997 | 0.910 |
| 426 | Training | Training | 3 | po | 0.5 | 1 | 1.7 | 4.2 | 0.190 | 0.074 | 1.597 | 2.510 |
| 427 | Training | Training | 3 | po | 0.5 | 1 | 1.2 | 2.2 | 0.220 | 0.081 | 1.926 | 1.424 |
| 428 | Training | Test | 3 | po | 0.5 | 1 | 1.0 | 1.3 | 0.300 | 0.094 | 1.127 | 1.086 |
| 429 | Training | Training | 10 | po | 1 | 4 | 7.1 | 9.5 | 0.030 | 0.008 | 0.221 | 0.213 |
| 430 | Training | Training | 3 | po | 0.5 | 1 | 2.4 | 1.1 | 0.490 | 0.210 | 0.218 | 0.292 |
| 431 | Training | Training | 3 | po | 0.5 | 1 | 26.2 | 5.2 | 0.540 | 0.104 | 1.500 | 0.750 |
| 432 | Test | Training | 3 | po | 0.5 | 1 | 7.7 | 25.4 | 0.150 | 0.071 | 0.326 | 1.083 |
| 433 | Training | Training | 3 | po | 0.5 | 1 | 4.8 | 2.8 | 0.210 | 0.117 | 0.306 | 0.348 |
| 434 | Test | Test | 3 | po | 0.5 | 1 | 4.9 | 3.1 | 0.400 | 0.162 | 1.346 | 1.464 |
| 435 | Training | Training | 3 | po | 0.5 | 1 | 1.9 | 0.9 | 0.590 | 0.283 | 0.706 | 0.377 |
| 436 | Training | Training | 10 | po | 0.5 | 1 | 40.1 | 14.0 | 0.630 | 0.185 | 0.152 | 0.110 |
| 437 | Training | Training | 3 | po | 0.5 | 1 | 10.1 | 19.0 | 0.310 | 0.131 | 0.256 | 0.300 |
| 438 | Test | Training | 3 | po | 0.5 | 1 | 80.0 | 16.2 | 0.880 | 0.270 | 0.178 | 0.176 |
| 439 | Training | Test | 3 | po | 0.5 | 1 | 2.5 | 2.4 | 0.530 | 0.195 | 1.105 | 1.249 |
| 440 | Training | Training | 3 | po | 1 | 4 | 6.2 | 1.7 | 0.100 | 0.018 | 1.096 | 1.235 |
| 441 | Training | Training | 3 | po | 0.5 | 1 | 1.3 | 0.8 | 0.140 | 0.105 | 0.729 | 0.215 |
| 442 | Training | Training | 3 | po | 0.5 | 1 | 2.6 | 2.8 | 0.230 | 0.056 | 1.830 | 1.701 |
| 443 | Training | Training | 3 | po | 0.5 | 1 | 1.6 | 0.9 | 0.520 | 0.207 | 1.639 | 3.409 |
| 444 | Training | Training | 100 | ip | 2 | 4 | 1.9 | 0.9 | 0.080 | 0.029 | 1.174 | 1.113 |
| 445 | Training | Test | 0.5 | iv | 0.083 | 0.25 | 8.1 | 0.9 | 0.030 | 0.013 | 0.482 | 0.324 |
| 446 | Training | Training | 3 | po | 0.5 | 1 | 16.1 | 5.0 | 0.090 | 0.123 | 0.129 | 0.118 |
| 447 | Training | Test | 3 | po | 0.5 | 1 | 2.7 | 1.1 | 0.600 | 0.341 | 0.461 | 0.410 |
| 448 | Training | Training | 3 | po | 0.5 | 1 | 16.9 | 2.0 | 0.270 | 0.165 | 0.338 | 0.368 |
| 449 | Test | Training | 10 | po | 0.5 | 1 | 4.1 | 1.5 | 0.130 | 0.022 | 2.224 | 1.856 |
| 450 | Training | Training | 3 | po | 0.5 | 1 | 8.3 | 2.6 | 0.300 | 0.135 | 0.389 | 0.397 |
| 451 | Training | Test | 3 | po | 0.5 | 1 | 9.0 | 1.3 | 0.190 | 0.077 | 0.230 | 0.351 |
| 452 | Training | Training | 1 | po | 0.5 | 1 | 2.8 | 2.7 | 0.400 | 0.129 | 0.637 | 0.612 |
| 453 | Test | Training | 10 | po | 0.5 | 1 | 3.7 | 1.6 | 0.110 | 0.024 | 1.819 | 1.685 |
| 454 | Training | Training | 3 | po | 1 | 4 | 7.0 | 1.6 | 0.140 | 0.024 | 1.017 | 1.325 |
| 455 | Training | Test | 3 | po | 0.5 | 1 | 3.3 | 2.2 | 0.370 | 0.217 | 0.632 | 0.633 |
| 456 | Test | Test | 1 | iv | 0.083 | 0.25 | 7.1 | 2.3 | 0.100 | 0.036 | 0.825 | 0.536 |
| 457 | Training | Training | 3 | po | 0.5 | 1 | 2.1 | 1.3 | 0.190 | 0.080 | 1.485 | 1.370 |
| 458 | Training | Test | 3 | po | 0.5 | 1 | 2.3 | 1.0 | 0.380 | 0.219 | 0.582 | 0.750 |
| 459 | Training | Training | 3 | po | 0.5 | 1 | 11.1 | 4.8 | 0.300 | 0.074 | 0.898 | 0.814 |
| 460 | Training | Training | 3 | po | 0.5 | 1 | 1.7 | 1.2 | 0.360 | 0.077 | 0.522 | 0.595 |
| 461 | Training | Training | 3 | po | 1 | 4 | 8.8 | 1.8 | 0.150 | 0.034 | 0.940 | 0.853 |
| 462 | Training | Test | 3 | po | 0.5 | 1 | 5.3 | 1.5 | 0.050 | 0.020 | 0.358 | 0.398 |
| 463 | Training | Test | 3 | po | 0.5 | 1 | 2.3 | 3.8 | 0.190 | 0.069 | 1.235 | 1.308 |
| 464 | Training | Training | 3 | po | 0.5 | 1 | 6.7 | 1.4 | 0.350 | 0.165 | 0.794 | 0.699 |
| 465 | Test | Training | 3 | po | 0.5 | 1 | 4.5 | 6.6 | 0.340 | 0.072 | 0.933 | 0.914 |
| 466 | Training | Training | 3 | po | 0.5 | 1 | 14.9 | 18.4 | 0.490 | 0.200 | 0.162 | 0.156 |
| 467 | Training | Test | 3 | po | 0.5 | 1 | 1.7 | 0.9 | 0.140 | 0.071 | 1.528 | 1.500 |
| 468 | Training | Training | 1 | po | 2 | 4 | 17.2 | 2.1 | 0.020 | 0.010 | 0.180 | 0.204 |
| 469 | Training | Test | 3 | po | 0.5 | 1 | 1.0 | 1.0 | 0.180 | 0.063 | 0.814 | 0.608 |
| 470 | Training | Test | 3 | po | 0.5 | 1 | 7.1 | 0.8 | 0.110 | 0.030 | 0.238 | 0.232 |
| 471 | Training | Training | 10 | po | 0.5 | 1 | 42.0 | 9.8 | 0.770 | 0.318 | 0.231 | 0.237 |
| 472 | Training | Test | 3 | po | 0.5 | 1 | 3.4 | 1.0 | 0.130 | 0.053 | 0.814 | 0.702 |
| 473 | Training | Training | 10 | po | 0.5 | 1 | 2.9 | 2.9 | 0.200 | 0.049 | 1.335 | 1.274 |
| 474 | Training | Training | 10 | po | 1 | 4 | 7.3 | 6.8 | 0.070 | 0.017 | 0.281 | 0.451 |
| 475 | Test | Test | 3 | po | 0.5 | 1 | 5.4 | 1.9 | 0.240 | 0.066 | 0.725 | 0.750 |
| 476 | Training | Test | 1 | iv | 0.083 | 0.25 | 6.1 | 1.5 | 0.050 | 0.052 | 0.462 | 0.268 |
| 477 | Test | Test | 1 | iv | 0.083 | 0.25 | 9.8 | 1.2 | 0.020 | 0.040 | 0.205 | 0.117 |
| 478 | Training | Test | 3 | po | 0.5 | 1 | 1.2 | 0.8 | 0.240 | 0.119 | 0.785 | 0.757 |
| 479 | Test | Test | 1 | iv | 0.083 | 0.25 | 12.6 | 2.9 | 0.050 | 0.051 | 0.190 | 0.103 |
| 480 | Training | Test | 3 | po | 0.5 | 1 | 3.9 | 1.1 | 0.400 | 0.142 | 1.084 | 1.117 |
| 481 | Test | Test | 1 | iv | 0.083 | 0.25 | 11.8 | 1.9 | 0.020 | 0.010 | 0.928 | 0.579 |
| 482 | Training | Test | 3 | po | 0.5 | 1 | 23.7 | 1.2 | 0.220 | 0.065 | 0.248 | 0.236 |
| 483 | Training | Test | 10 | po | 0.5 | 1 | 17.3 | 9.4 | 0.750 | 0.245 | 0.490 | 0.706 |
| 484 | Training | Test | 3 | po | 0.5 | 1 | 1.9 | 1.9 | 0.170 | 0.100 | 0.316 | 0.390 |
| 485 | Training | Test | 3 | po | 0.5 | 1 | 2.8 | 0.8 | 0.320 | 0.229 | 0.188 | 0.208 |
| 486 | Training | Test | 3 | po | 0.5 | 1 | 53.5 | 7.0 | 0.150 | 0.128 | 0.069 | 0.064 |
| 487 | Test | Test | 3 | po | 0.5 | 1 | 4.5 | 1.8 | 0.440 | 0.199 | 0.840 | 1.007 |
| 488 | Training | Test | 3 | po | 0.5 | 1 | 10.1 | 0.9 | 0.030 | 0.013 | 0.593 | 0.472 |
| 489 | Test | Test | 1 | iv | 0.083 | 0.25 | 26.9 | 1.8 | 0.150 | 0.098 | 0.239 | 0.130 |
| 490 | Training | Test | 10 | po | 0.5 | 1 | 3.1 | 9.1 | 0.400 | 0.223 | 0.476 | 0.486 |
| 491 | Training | Training | 3 | ip | 0.083 | 0.25 | 2.5 | 2.6 | 0.110 | 0.027 | 0.330 | 0.445 |
| 492 | Training | Training | 3 | po | 0.5 | 1 | 9.1 | 4.5 | 0.250 | 0.146 | 0.427 | 0.697 |
| 493 | Training | Training | 10 | sc | 0.5 | 2 | 50.7 | 1.6 | 0.180 | 0.157 | 0.085 | 0.068 |
| 494 | Training | Training | 10 | sc | 0.5 | 2 | 6.6 | 0.8 | 0.130 | 0.030 | 1.021 | 1.318 |
| 495 | Test | Training | 3 | po | 1 | 2 | 2.3 | 1.6 | 0.390 | 0.135 | 0.351 | 0.324 |
| 496 | Training | Training | 3 | po | 1 | 4 | 5.9 | 1.8 | 0.140 | 0.058 | 0.754 | 0.758 |
| 497 | Training | Training | 10 | po | 0.5 | 1 | 1.0 | 1.2 | 0.560 | 0.191 | 1.682 | 1.616 |
| 498 | Training | Training | 3 | po | 0.5 | 1 | 1.8 | 1.0 | 0.290 | 0.107 | 1.698 | 1.396 |
| 499 | Training | Training | 1 | po | 1 | 4 | 13.6 | 3.4 | 0.090 | 0.009 | 2.669 | 3.486 |
| 500 | Training | Training | 10 | po | 0.5 | 1 | 48.9 | 11.5 | 0.420 | 0.084 | 0.699 | 1.071 |
| 501 | Training | Training | 3 | ip | 0.5 | 1 | 9.3 | 6.9 | 0.180 | 0.064 | 0.503 | 0.482 |
| 502 | Test | Training | 3 | po | 0.5 | 1 | 4.1 | 3.6 | 0.710 | 0.214 | 0.858 | 0.517 |
| 503 | Training | Training | 3 | ip | 0.083 | 0.25 | 1.8 | 1.0 | 0.150 | 0.086 | 0.347 | 0.432 |
| 504 | Training | Training | 2.5 | ip | 0.083 | 0.25 | 1.9 | 1.3 | 0.490 | 0.025 | 1.740 | 4.766 |
| 505 | Training | Training | 10 | sc | 0.5 | 2 | 47.9 | 1.0 | 0.270 | 0.160 | 0.118 | 0.092 |
| 506 | Training | Training | 10 | sc | 0.5 | 2 | 14.7 | 1.0 | 0.330 | 0.114 | 0.401 | 0.429 |
| 507 | Training | Training | 10 | ip | 0.25 | 0.5 | 17.9 | 1.8 | 0.220 | 0.058 | 0.397 | 0.418 |
| 508 | Training | Training | 1 | iv | 0.25 | 0.5 | 7.8 | 1.4 | 0.200 | 0.098 | 0.693 | 0.614 |
| 509 | Test | Training | 3 | po | 0.5 | 1 | 3.6 | 1.4 | 0.050 | 0.042 | 0.267 | 0.230 |
| 510 | Training | Training | 3 | po | 0.5 | 1 | 5.3 | 2.0 | 0.190 | 0.080 | 0.605 | 0.750 |
| 511 | Training | Training | 10 | po | 0.5 | 1 | 150.9 | 12.7 | 0.370 | 0.159 | 0.305 | 0.333 |
| 512 | Training | Training | 10 | sc | 0.5 | 2 | 21.4 | 0.8 | 0.470 | 0.242 | 0.124 | 0.172 |
| 513 | Training | Training | 0.2 | iv | 0.5 | 2 | 17.6 | 1.6 | 0.070 | 0.058 | 0.781 | 0.748 |
| 514 | Training | Training | 3 | po | 0.5 | 1 | 5.0 | 2.6 | 0.180 | 0.104 | 0.515 | 0.434 |
| 515 | Training | Training | 3 | po | 0.5 | 1 | 149.4 | 8.1 | 0.930 | 0.443 | 0.057 | 0.040 |
| 516 | Training | Training | 3 | po | 0.5 | 1 | 11.4 | 8.6 | 0.400 | 0.329 | 0.104 | 0.096 |
| 517 | Test | Training | 1 | po | 1 | 2 | 4.1 | 0.9 | 0.020 | 0.002 | 2.975 | 3.337 |
| 518 | Training | Training | 10 | po | 0.5 | 1 | 43.5 | 8.2 | 0.800 | 0.195 | 0.313 | 0.384 |
| 519 | Training | Training | 3 | po | 0.5 | 1 | 1.4 | 0.7 | 0.150 | 0.054 | 1.535 | 1.557 |
| 520 | Training | Training | 0.2 | iv | 0.5 | 2 | 2.3 | 5.9 | 0.150 | 0.043 | 0.000 | 0.161 |
| 521 | Test | Training | 10 | sc | 1 | 3 | 20.2 | 1.0 | 0.370 | 0.237 | 0.132 | 0.138 |
| 522 | Training | Training | 3 | po | 1 | 2 | 5.7 | 1.7 | 0.250 | 0.085 | 0.380 | 0.347 |
| 523 | Training | Training | 3 | ip | 0.25 | 0.5 | 13.0 | 2.6 | 0.450 | 0.297 | 0.522 | 0.363 |
| 524 | Test | Training | 3 | po | 0.5 | 1 | 33.1 | 10.5 | 0.600 | 0.232 | 0.333 | 0.384 |
| 525 | Training | Training | 10 | po | 0.5 | 1 | 8.6 | 0.7 | 0.790 | 0.215 | 4.262 | 4.283 |
| 526 | Training | Training | 3 | po | 0.5 | 1 | 32.0 | 3.9 | 0.580 | 0.132 | 0.143 | 0.167 |
| 527 | Training | Training | 3 | iv | 0.083 | 0.5 | 35.7 | 0.7 | 0.330 | 0.272 | 0.160 | 0.223 |
| 528 | Training | Training | 1 | iv | 0.083 | 0.5 | 37.9 | 0.8 | 0.510 | 0.266 | 0.209 | 0.187 |
| 529 | Training | Training | 10 | ip | 0.25 | 0.5 | 4.3 | 0.7 | 0.070 | 0.020 | 1.172 | 0.790 |
| 530 | Training | Training | 3 | iv | 0.25 | 0.5 | 5.5 | 1.1 | 0.210 | 0.053 | 0.824 | 0.746 |
| 531 | Test | Training | 3 | po | 1 | 2 | 12.6 | 1.6 | 0.420 | 0.123 | 0.356 | 0.361 |
| 532 | Training | Training | 10 | po | 0.5 | 1 | 98.9 | 1.5 | 0.810 | 0.340 | 0.155 | 0.225 |
| 533 | Test | Training | 3 | po | 0.5 | 1 | 45.0 | 12.5 | 0.520 | 0.198 | 0.083 | 0.093 |
| 534 | Training | Training | 3 | iv | 0.083 | 0.5 | 26.3 | 0.7 | 0.180 | 0.228 | 0.249 | 0.286 |
| 535 | Training | Training | 10 | sc | 0.5 | 2 | 15.1 | 0.7 | 0.300 | 0.126 | 0.240 | 0.443 |
| 536 | Test | Training | 3 | po | 1 | 2 | 1.5 | 1.1 | 0.230 | 0.095 | 0.356 | 0.361 |
| 537 | Training | Training | 3 | ip | 0.25 | 0.5 | 12.1 | 2.3 | 0.610 | 0.244 | 0.612 | 0.784 |
| 538 | Training | Training | 3 | po | 0.5 | 1 | 20.3 | 6.1 | 0.580 | 0.171 | 0.376 | 0.083 |
| 539 | Training | Training | 10 | po | 0.5 | 1 | 2.9 | 0.6 | 0.090 | 0.040 | 1.674 | 1.561 |
| 540 | Test | Training | 10 | sc | 0.5 | 2 | 12.9 | 0.9 | 0.260 | 0.157 | 0.086 | 0.407 |
| 541 | Training | Training | 3 | sc | 0.5 | 2 | 22.4 | 0.8 | 0.250 | 0.137 | 0.400 | 0.360 |
| 542 | Training | Training | 10 | po | 0.5 | 1 | 1.7 | 0.6 | 0.010 | 0.021 | 0.121 | 0.123 |
| 543 | Training | Training | 10 | po | 0.5 | 1 | 2.5 | 1.0 | 0.270 | 0.298 | 0.366 | 0.334 |
| 544 | Training | Training | 3 | po | 0.5 | 1 | 27.5 | 3.3 | 0.410 | 0.086 | 0.922 | 1.195 |
| 545 | Training | Training | 1 | po | 2 | 4 | 14.4 | 1.0 | 0.130 | 0.053 | 0.479 | 0.514 |
| 546 | Training | Training | 3 | po | 1 | 2 | 0.8 | 1.0 | 0.500 | 0.051 | 9.985 | 12.771 |
| 547 | Training | Training | 3 | po | 0.5 | 1 | 16.1 | 5.2 | 0.210 | 0.094 | 0.145 | 0.156 |
| 548 | Test | Training | 10 | sc | 0.5 | 2 | 63.4 | 2.3 | 0.390 | 0.152 | 0.061 | 0.075 |
| 549 | Training | Training | 10 | po | 0.5 | 2 | 9.9 | 1.1 | 0.120 | 0.150 | 0.189 | 0.081 |
| 550 | Training | Training | 10 | ip | 0.25 | 0.5 | 4.5 | 1.4 | 0.250 | 0.090 | 0.493 | 0.637 |
| 551 | Training | Training | 10 | po | 0.5 | 1 | 4.1 | 1.0 | 0.350 | 0.053 | 6.060 | 5.704 |
| 552 | Training | Training | 3 | po | 1 | 2 | 0.9 | 0.6 | 0.430 | 0.026 | 14.893 | 21.213 |
| 553 | Training | Training | 1 | po | 2 | 4 | 66.0 | 2.2 | 0.040 | 0.032 | 0.054 | 0.049 |
| 554 | Training | Training | 3 | sc | 0.5 | 2 | 13.7 | 0.9 | 0.400 | 0.182 | 0.469 | 0.413 |
| 555 | Training | Training | 10 | po | 0.5 | 1 | 20.6 | 1.3 | 0.250 | 0.049 | 0.913 | 1.637 |
| 556 | Training | Training | 10 | ip | 0.25 | 0.5 | 6.6 | 1.9 | 0.250 | 0.079 | 0.925 | 0.778 |
| 557 | Training | Training | 10 | po | 0.5 | 1 | 4.8 | 0.8 | 0.290 | 0.050 | 0.650 | 0.484 |
| 558 | Training | Training | 1 | iv | 0.083 | 0.5 | 3.0 | 0.6 | 0.020 | 0.007 | 2.248 | 2.283 |
| 559 | Training | Test | 1 | po | 0.5 | 1 | 88.2 | 2.9 | 0.210 | 0.088 | 0.050 | 0.054 |
| 560 | Training | Test | 3 | po | 0.5 | 1 | 1.6 | 0.7 | 0.330 | 0.199 | 0.233 | 0.368 |
| 561 | Test | Test | 1 | iv | 0.083 | 0.25 | 10.8 | 2.7 | 0.040 | 0.056 | 0.276 | 0.231 |
| 562 | Test | Test | 1 | iv | 0.083 | 0.25 | 46.7 | 5.6 | 0.060 | 0.031 | 0.204 | 0.119 |
| 563 | Training | Test | 1 | po | 0.5 | 1 | 3.4 | 1.0 | 0.010 | 0.017 | 0.261 | 0.270 |
| 564 | Training | Test | 3 | po | 0.5 | 1 | 2.1 | 1.6 | 0.180 | 0.056 | 0.835 | 1.003 |
| 565 | Training | Test | 3 | po | 0.5 | 1 | 4.7 | 0.9 | 0.250 | 0.081 | 1.284 | 1.023 |
| 566 | Test | Test | 3 | po | 0.5 | 1 | 12.3 | 2.0 | 0.680 | 0.126 | 2.575 | 2.336 |
| 567 | Training | Test | 1 | iv | 0.083 | 0.25 | 2.8 | 1.1 | 0.010 | 0.005 | 0.811 | 0.676 |
| 568 | Training | Test | 3 | po | 0.5 | 1 | 1.7 | 0.7 | 0.270 | 0.126 | 0.208 | 0.224 |
| 569 | Test | Test | 3 | po | 0.5 | 1 | 52.0 | 1.2 | 0.080 | 0.039 | 0.041 | 0.042 |
| 570 | Training | Test | 1 | iv | 0.083 | 0.25 | 9.9 | 1.9 | 0.020 | 0.012 | 0.782 | 0.413 |
| 571 | Training | Test | 3 | po | 0.5 | 1 | 3.5 | 1.3 | 0.020 | 0.003 | 1.295 | 1.197 |
| 572 | Training | Test | 1 | iv | 0.083 | 0.25 | 12.4 | 0.6 | 0.070 | 0.039 | 0.347 | 0.187 |
| 573 | Training | Training | 10 | po | 0.5 | 1 | 6.1 | 3.1 | 0.230 | 0.081 | 0.564 | 0.595 |
| 574 | Training | Test | 3 | po | 0.5 | 1 | 15.6 | 3.0 | 0.670 | 0.139 | 1.905 | 1.820 |
| 575 | Training | Training | 100 | ip | 1 | 4 | 6.6 | 0.8 | 0.010 | 0.002 | 2.620 | 2.737 |
| 576 | Training | Training | 100 | ip | 1 | 4 | 2.5 | 1.8 | 0.010 | 0.002 | 0.746 | 2.083 |
| 577 | Test | Training | 1 | po | 0.5 | 1 | 11.1 | 3.0 | 0.170 | 0.118 | 0.229 | 0.261 |
| 578 | Training | Training | 1 | po | 0.5 | 1 | 6.9 | 2.4 | 0.140 | 0.070 | 0.203 | 0.278 |
| 579 | Training | Test | 3 | po | 0.5 | 1 | 7.0 | 2.2 | 0.500 | 0.211 | 0.839 | 0.565 |
| 580 | Training | Test | 3 | po | 0.5 | 1 | 4.7 | 2.1 | 0.520 | 0.216 | 0.528 | 0.361 |
| 581 | Training | Test | 3 | po | 0.5 | 1 | 16.0 | 2.8 | 0.340 | 0.145 | 0.405 | 0.386 |
| 582 | Training | Test | 3 | po | 0.5 | 1 | 2.4 | 1.0 | 0.560 | 0.286 | 0.322 | 0.395 |
| 583 | Test | Test | 3 | po | 0.5 | 1 | 1.8 | 0.5 | 0.450 | 0.138 | 0.481 | 0.440 |
| 584 | Test | Test | 1 | iv | 0.083 | 0.25 | 6.6 | 0.6 | 0.040 | 0.043 | 0.657 | 0.245 |
| 585 | Training | Test | 1 | iv | 0.083 | 0.25 | 5.7 | 0.8 | 0.020 | 0.007 | 1.446 | 0.728 |
| 586 | Training | Test | 1 | po | 0.5 | 1 | 16.8 | 1.1 | 0.240 | 0.064 | 0.129 | 0.104 |
| 587 | Training | Test | 3 | po | 0.5 | 1 | 11.6 | 1.7 | 0.040 | 0.014 | 0.224 | 0.239 |
| 588 | Training | Test | 1 | iv | 0.083 | 0.25 | 5.0 | 1.4 | 0.030 | 0.010 | 0.637 | 0.549 |
| 589 | Training | Test | 3 | po | 0.5 | 1 | 5.5 | 1.4 | 0.060 | 0.016 | 0.360 | 0.375 |
| 590 | Training | Test | 3 | po | 0.5 | 1 | 12.7 | 1.0 | 0.230 | 0.097 | 0.289 | 0.370 |
| 591 | Training | Test | 3 | po | 0.5 | 1 | 31.9 | 8.2 | 0.130 | 0.054 | 0.104 | 0.168 |
| 592 | Training | Training | 3 | po | 1 | 2 | 5.0 | 3.9 | 0.340 | 0.169 | 0.317 | 0.387 |
| 593 | Training | Training | 0.3 | po | 4 | 24 | 43.4 | 22.7 | 0.020 | 0.011 | 0.082 | 0.035 |
| 594 | Training | Training | 3 | po | 1 | 2 | 24.4 | 3.8 | 0.010 | 0.009 | 0.056 | 0.095 |
| 595 | Training | Training | 3 | po | 0.5 | 1 | 2.1 | 2.1 | 0.030 | 0.015 | 0.338 | 0.329 |
| 596 | Training | Training | 3 | po | 0.5 | 1 | 8.7 | 5.2 | 0.030 | 0.018 | 0.074 | 0.082 |
| 597 | Training | Training | 10 | ip | 1 | 2 | 1.2 | 0.8 | 0.070 | 0.025 | 2.115 | 1.868 |
| 598 | Training | Training | 3 | po | 0.5 | 1 | 6.1 | 9.4 | 0.450 | 0.105 | 0.922 | 1.292 |
| 599 | Training | Training | 3 | po | 0.5 | 1 | 26.1 | 35.2 | 0.260 | 0.060 | 0.228 | 0.256 |
| 600 | Test | Training | 3 | po | 1 | 2 | 1.3 | 1.2 | 0.110 | 0.050 | 0.631 | 0.485 |
| 601 | Training | Training | 3 | po | 1 | 2 | 0.6 | 2.3 | 0.020 | 0.009 | 0.733 | 0.737 |
| 602 | Training | Training | 1 | po | 0.5 | 1 | 13.7 | 9.6 | 0.150 | 0.035 | 0.469 | 0.321 |
| 603 | Training | Training | 1 | po | 0.5 | 1 | 9.8 | 4.7 | 0.310 | 0.145 | 0.790 | 0.574 |
| 604 | Training | Training | 3 | po | 1 | 2 | 1.2 | 0.8 | 0.140 | 0.036 | 0.941 | 0.817 |
| 605 | Test | Training | 3 | po | 0.5 | 1 | 3.8 | 2.5 | 0.260 | 0.126 | 0.972 | 0.904 |
| 606 | Training | Training | 3 | po | 0.5 | 1 | 3.2 | 2.2 | 0.210 | 0.115 | 1.589 | 1.339 |
| 607 | Training | Training | 3 | po | 1 | 4 | 10.1 | 2.5 | 0.160 | 0.041 | 1.175 | 1.217 |
| 608 | Training | Training | 3 | po | 0.5 | 1 | 28.3 | 5.7 | 0.050 | 0.023 | 0.023 | 0.028 |
| 609 | Training | Training | 1 | po | 1 | 2 | 1.2 | 0.6 | 0.030 | 0.006 | 3.367 | 2.911 |
| 610 | Training | Test | 3 | po | 0.5 | 1 | 1.3 | 0.87 | 0.290 | 0.179 | 0.783 | 0.783 |
| 611 | Test | Test | 3 | po | 0.5 | 1 | 1.9 | 1.3 | 0.340 | 0.140 | 0.684 | 0.870 |
| 612 | Test | Test | 1 | iv | 0.5 | 1 | 51 | 11 | 0.250 | 0.032 | 0.400 | 0.444 |
| 613 | Training | Test | 3 | iv | 0.5 | 1 | 43 | 46 | 0.300 | 0.150 | 0.025 | 0.026 |
| 614 | Training | Test | 1 | iv | 0.5 | 1 | 10.45 | 2.3 | 0.070 | 0.011 | 0.291 | 0.305 |
| 615 | Test | Test | 1 | iv | 0.5 | 1 | 22 | 3.8 | 0.050 | 0.033 | 0.197 | 0.304 |
| 616 | Training | Test | 1 | iv | 0.5 | 1 | 45 | 30 | 0.060 | 0.012 | 0.060 | 0.110 |
| 617 | Training | Test | 1 | iv | 0.5 | 1 | 23 | 31 | 0.080 | 0.056 | 0.072 | 0.056 |
| 618 | Training | Test | 1 | iv | 0.5 | 1 | 47 | 42 | 0.090 | 0.060 | 0.074 | 0.110 |
| 619 | Test | Test | 1 | iv | 0.5 | 1 | 66 | 4.8 | 0.030 | 0.009 | 0.239 | 0.154 |
| 620 | Training | Test | 1 | iv | 0.5 | 1 | 26 | 11 | 0.230 | 0.077 | 0.226 | 0.109 |
| 621 | Training | Test | 1 | iv | 0.5 | 1 | 19 | 2.2 | 0.070 | 0.036 | 0.236 | 0.325 |
| 622 | Test | Test | 1 | iv | 0.5 | 1 | 39 | 19 | 0.290 | 0.045 | 0.608 | 1.205 |
| 623 | Training | Test | 1 | iv | 0.5 | 1 | 28 | 8.8 | 0.020 | 0.020 | 0.114 | 0.091 |
| 624 | Training | Test | 1 | iv | 0.5 | 1 | 21 | 2.2 | 0.050 | 0.022 | 0.213 | 0.189 |
| 625 | Test | Test | 1 | iv | 0.5 | 1 | 47 | 1.4 | 0.090 | 0.031 | 0.535 | 0.596 |
| 626 | Training | Test | 1 | iv | 0.5 | 1 | 53 | 26 | 0.290 | 0.075 | 0.167 | 0.192 |
| 627 | Training | Test | 1 | iv | 0.5 | 1 | 75 | 12 | 0.140 | 0.051 | 0.075 | 0.077 |
| 628 | Training | Test | 3 | po | 0.5 | 1 | 3.7 | 1.1 | 0.070 | 0.045 | 0.183 | 0.231 |
| 629 | Training | Test | 1 | iv | 0.5 | 1 | 13 | 3.7 | 0.160 | 0.044 | 0.566 | 0.543 |
| 630 | Test | Test | 1 | iv | 0.5 | 1 | 10 | 1.7 | 0.040 | 0.013 | 0.501 | 0.459 |
| 631 | Test | Test | 1 | iv | 0.5 | 1 | 52 | 11 | 0.150 | 0.035 | 0.288 | 0.285 |
| 632 | Test | Test | 3 | iv | 0.5 | 1 | 110 | 23 | 0.380 | 0.151 | 0.043 | 0.040 |
| 633 | Training | Test | 3 | iv | 0.5 | 1 | 110 | 21 | 0.410 | 0.145 | 0.030 | 0.010 |
| 634 | Training | Test | 3 | iv | 0.5 | 1 | 8.3 | 14 | 0.370 | 0.108 | 0.108 | 0.621 |
| 635 | Training | Test | 3 | po | 0.5 | 1 | 7.25 | 8 | 0.110 | 0.017 | 0.345 | 0.248 |
| 636 | Training | Test | 1 | po | 0.5 | 1 | 11 | 6.9 | 0.120 | 0.018 | 0.653 | 0.439 |
| 637 | Training | Test | 1 | po | 0.5 | 1 | 7.8 | 7.2 | 0.120 | 0.019 | 0.771 | 0.550 |
| 638 | Training | Test | 3 | po | 0.5 | 1 | 2.1 | 2.3 | 0.060 | 0.010 | 0.919 | 0.518 |
| 639 | Training | Test | 1 | iv | 0.5 | 1 | 120 | 25 | 0.120 | 0.062 | 0.050 | 0.077 |
| 640 | Training | Test | 1 | iv | 0.5 | 1 | 37 | 52 | 0.310 | 0.042 | 0.161 | 0.321 |
|  |  |  |  |  |  |  |  |  |  |  |  |  |

**Table S6. Summary of Hyperparameters in GPOPT model**

|  |  |  |  |  |
| --- | --- | --- | --- | --- |
| Model | Split | θ1 | θ2 | θ3 |
| ER in MDR1 | Cluster | 66.2 | 48.9 | 0.980 |
|  | Time | 65.8 | 48.6 | 0.980 |
| ER in BCRP | Cluster | 59.7 | 44.1 | 0.980 |
|  | Time | 57.6 | 42.6 | 0.980 |
| K_p,uu,brain_ | Cluster | 45.1 | 33.3 | 0.760 |
|  | Time | 49.1 | 36.6 | 0.980 |
